# Multimodal Cross‐Attentive Graph‐Based Framework for Predicting In Vivo Endocrine Disruptors

**DOI:** 10.1002/advs.202519897

**Published:** 2026-02-15

**Authors:** Eder Soares de Almeida Santos, Gustavo Felizardo Santos Sandes, Artur Christian Garcia da Silva, Holli‐Joi Martin, Eugene N. Muratov, Rodolpho de Campos Braga, Bruno Junior Neves

**Affiliations:** ^1^ Laboratory of Cheminformatics, Faculty of Pharmacy Universidade Federal de Goiás Goiás Brazil; ^2^ Laboratory of Education and Research in In Vitro Toxicology, Faculty of Pharmacy Universidade Federal de Goiás Goiás Brazil; ^3^ Laboratory for Molecular Modeling, UNC Eshelman School of Pharmacy University of North Carolina at Chapel Hill Chapel Hill North Carolina USA; ^4^ InsilicAll Ltda. São Paulo Brazil

**Keywords:** adverse outcome pathway, androgen, deep learning, endocrine disruption, estrogen

## Abstract

Endocrine hazard assessment needs models that are accurate and mechanistically transparent. We present a multimodal cross‐attentive graph framework that fuses molecular graphs with adverse‐outcome‐pathway (AOP)–anchored assay signals to predict organism‐level outcomes in the organisation for economic co‐operation and development (OECD) Hershberger and uterotrophic assays. In Tier‐1, multitask graph neural networks (GNNs) learn estrogen and androgen receptor molecular‐initiating and key events across 46 in vitro ToxCast/Tox21 assays. In Tier‐2, a cross‐attentive multimodal GNN integrates Tier‐1 pathway signals with molecular graphs, yielding high predictive performance for both the in vivo Hershberger (AUROC = 0.97 ± 0.014) and uterotrophic (AUROC = 0.97 ± 0.008) assays. Retrospective analysis of literature compounds showed 88% concordance (Hershberger 15/18; uterotrophic 23/26). Bidirectional cross‐attention highlights associations between molecular substructures and pathway‐level assay nodes, while counterfactual perturbations rank assays and structural motifs most influential for each decision. The framework couple's high accuracy with assay‐traceable explanations, supporting targeted testing within the integrated approaches.

## Graphical abstract

A multimodal cross‐attentive graph neural network integrates molecular graphs with androgen and estrogen adverse outcome pathway (AOP)–anchored in vitro assay signals to predict in vivo endocrine disruption. By fusing information on Tier‐1 AOP logits with chemical structures, the framework achieves high accuracy and provides assay‐traceable, mechanistic explanations for Hershberger and uterotrophic outcomes.

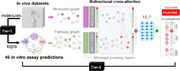

## 1 Introduction

Endocrine‐disrupting chemicals (EDCs) comprise a group of exogenous substances that interfere with hormone signaling pathways, particularly through estrogen (ER) and androgen (AR) receptor mechanisms [1, 2], leading to well‐documented adverse outcomes ranging from reproductive disorders to hormone‐dependent cancers [3, 4, 5, 6, 7]. International evaluations and economic analyses estimate that such exposures contribute to widespread disease at the population level and generate annual costs amounting to hundreds of billions of euros in the European Union [8].

Assessment of chemical hazards has historically relied on in vivo assays established under the Organization for Economic Co‐operation and Development (OECD) test guidelines to capture AR‐ and ER‐mediated mechanisms. The Hershberger assay (OECD test no. 441), conducted in castrated male rats supplemented with testosterone propionate, provides direct evidence of AR agonism or antagonism through changes in male accessory sex organ weights. Similarly, the uterotrophic assay (OECD test no. 440), performed in immature or ovariectomized female rodents, monitors ER modulation by measuring uterine weight and histological changes. While these assays serve as regulatory benchmarks for identifying endocrine activity, they have limited throughput, high costs, and ethical concerns associated with the use of animals [9].

In addition, high‐throughput screening strategies have been established for assessing EDCs. The U.S. Environmental Protection Agency's (EPA) ToxCast program and the interagency Tox21 collaboration have generated extensive in vitro bioactivity data across ∼10 000 chemicals [10, 11, 12], including dedicated assay batteries for AR‐ [13] and ER‐mediated [14] adverse outcome pathways (AOPs). These resources provide unprecedented mechanistic insight for regulatory prioritization but remain constrained by limited metabolic competence and the challenge of translating pathway perturbations into complex in vivo outcomes [15, 16]. Building on these efforts, the U.S. EPA implemented the Endocrine Disruptor Screening Program (EDSP) to determine potential endocrine effects in humans and wildlife. The program was structured in two tiers: Tier‐1 was designed to identify potential EDCs through a battery of in vitro and in vivo assays (including the OECD Hershberger and uterotrophic tests); and Tier‐2 was intended to characterize dose‐response relationships and adverse outcomes in more comprehensive animal studies [16, 17]. Despite its ambitious scope, the EDSP has progressed slowly, with Tier‐1 testing completed for only a limited number of chemicals.

Considering these limitations, computational toxicology has emerged as a promising approach to enhance the throughput of chemical assessments [18]. Within computational toxicology, machine learning approaches leverage large‐scale biological, chemical, and mechanistic data to uncover non‐linear patterns underlying endocrine disruption [19, 20, 21, 22, 23, 24, 25], thereby enabling regulatory prioritization while reducing reliance on animal testing. These approaches have been increasingly adopted into Integrated Approaches to Testing and Assessment (IATA) frameworks as complementary tools to conventional assays, offering a scalable strategy to screen the thousands of chemicals that remain untested for endocrine activity [26, 27].

In recent years, graph neural networks (GNNs) [28, 29] have expanded the landscape of predictive toxicology. These models rely on molecular graphs, in which nodes represent atoms, edges represent bonds, and attributes (e.g., atom type, chirality, and bond order) are encoded as features. GNNs offer adaptable architectures for modeling complex molecular interactions and endocrine‐related responses. However, current applications in computational toxicology rely primarily on molecular structure information, with underutilization of biological pathway data. Despite promising advances, these studies remain limited in number and scope, focusing mainly on in vitro AR‐ and ER‐mediated effects [30, 31, 32]. Extending these efforts toward more comprehensive predictions, particularly those aligned with organism‐level outcomes, requires integrative modeling strategies that combine molecular graph representations with adverse outcome pathways and assay‐derived information. Such approaches enable the capture of receptor‐level interactions while also accounting for downstream biological responses.

In this study, we introduce a multimodal cross‐attentive graph framework that uniquely integrates chemical structure information with AOP‐based predictions to model in vivo endocrine disruption. The approach was developed in two tiers: first, multitask GNNs were trained on 46 in vitro assays from the ToxCast/Tox21 program to capture AR‐ and ER‐mediated molecular initiating events (MIEs) and key events (KEs); second, the resulting predictions and logits—rather than the raw in vitro labels—were embedded within a pathway graph and integrated with molecular graphs through bidirectional cross‐attentive multimodal GNNs to predict outcomes of the OECD in vivo Hershberger (AUROC = 0.97 ± 0.014) and uterotrophic (AUROC = 0.97 ± 0.008) assays.

Beyond predictive performance, the multimodal framework provides explainability through bidirectional cross‐attention, which links molecular substructures to pathway assays and, conversely, pathway nodes to their most influential molecular features. This dual mapping elucidates how structural motifs propagate into AR‐ and ER‐mediated perturbations. As an additional interpretability strategy, counterfactual perturbations were employed to generate alternative scenarios, enabling the evaluation of how molecular modifications or pathway disruptions may alter downstream outcomes.

## 2 Materials and Methods

### 2.1 Data Collection and Curation

Initially, we assembled a dataset comprising 10 000 chemical entries from the U.S. EPA ToxCast/Tox21 program (in vitroDB v4.1), encompassing 46 in vitro assays relevant to androgen receptor (AR)– and estrogen receptor (ER)–mediated adverse outcome pathways (AOPs). In parallel, we curated a total of 957 in vivo chemical entries derived from rodent endocrine assays, including 499 compounds evaluated using the Hershberger assay [33] and 458 compounds assessed in the uterotrophic assay [34]. Subsequently, chemical structures in simplified molecular input line entry system (SMILES) format and their outcomes were curated according to the protocol established by Fourches et al., [35, 36]. The process involved standardization of functional groups and aromatic systems and removal of salts, mixtures, polymers, and organometallics using RDKit v.2024.4.5. Furthermore, we performed the analysis and exclusion of duplicates as follows: (i) if duplicates presented discordance in experimental outcomes (e.g., active vs. inactive), both entries would be excluded; and (ii) if the reported outcomes of the duplicates were the same, one entry would be retained in the dataset and the other excluded. After curation, the in vitro dataset comprised 7956 compounds, whereas the in vivo datasets contained 136 compounds for the Hershberger assay and 118 compounds for the uterotrophic assay. A detailed description of each assay is provided in Table S1.

### 2.2 Data Augmentation and Partitioning Scheme

To mitigate in vivo data scarcity, a structured data augmentation procedure was employed to incorporate additional putative compounds from the ToxCast/Tox21 database. Specifically, we added 717 compounds to the Hershberger dataset and 747 compounds to the uterotrophic dataset, prioritizing hard negatives, i.e., active compounds in parallel biological pathways or unrelated assay endpoints that remain consistently inactive in the AR‐ or ER‐mediated MIE and KE5 assays. Finally, the in vitro and in vivo datasets were randomly partitioned into training, validation, and test sets using an 8:1:1 ratio. This procedure was repeated across five independent splits generated with different random seeds. For each split, models were trained on the training set, hyperparameters were tuned on the validation set, and final performance was reported on the held‐out test set to provide an unbiased estimate of predictive accuracy. To avoid information leakage, we additionally employed the Murcko scaffold split approach on both in vitro and in vivo datasets [37]. However, due to the extreme sparsity of actives in these datasets, scaffold splits frequently yielded validation/test folds with zero or near‐zero positives (e.g., the uterotrophic scaffold split produced 0 actives in both validation and test), rendering hyperparameter tuning and metric estimation unstable. Split compositions for random and scaffold schemes are summarized in Table S2.

### 2.3 Tier‐1 Models

Tier‐1 models were developed to predict the outcomes of 46 in vitro assays encompassing AR‐ and ER‐mediated MIEs and KEs, using molecular graphs as feature representations.

#### 2.3.1 Molecular Graphs

Undirected molecular graphs were constructed from SMILES strings using RDKit v.2024.4.5, representing atoms as nodes and bonds as edges. Detailed specifications for all atom and bond features are provided in Table S3. Briefly, atom features comprised one‐hot encodings of atomic number, degree, formal charge, hybridization, and aromaticity. Bond features were encoded through one‐hot representations of bond type, conjugation, ring membership, stereochemistry, and chirality.

#### 2.3.2 Architecture

The Message Passing Neural Network (MPNN) [38], Graph Attention Network (GAT) [39], and Graph Isomorphism Network (GIN) [40] architectures were refined by systematically incorporating skip connections, virtual nodes, and Jumping Knowledge (JK) mechanisms to enhance chemical semantics. All graph‐based models additionally integrated GraphNorm normalization [41] at each message‐passing layer to stabilize training dynamics and improve representation consistency across graphs of varying sizes. Furthermore, the Attentive Fingerprint (AttentiveFP) [42] architecture was employed without modification, as it inherently incorporates multiscale mechanisms analogous to those introduced in the optimized models. Skip connections were incorporated to mitigate over‐smoothing and gradient degradation during deep message‐passing as follows:

$$h^{\left(\ell \right)}\leftarrow \;h^{\left(\ell \right)}+h^{\left(\ell -1\right)}$$

where $h^{\left(\ell -1\right)}$, $h^{\left(\ell \right)}\;\in \;R^{d}$ are input and output features at layer ℓ. This additive transform acts as an identity initialization, preserving the gradient flow.

The virtual node mechanism was incorporated into each molecular graph to enrich chemical semantics by forging long‐range connections among pharmacophoric and structural features. This node is initialized with a learnable embedding by $h_{virt}^{\left(0\right)}\in \;R^{d}$ and interacts with all nodes by contributing additively to their representations at each message‐passing layer. For a given graph *G*  = (*V*,  *E*) , the representation of each atom ν∈V at layer ℓ is modified as follows:

$$h_{v}^{\left(\ell \right)}\;\leftarrow \;h_{v}^{\left(\ell \right)}+h_{virt}^{\left(\ell \right)}$$

where $h_{v}^{\left(\ell \right)}$ is the atom's representation at layer ℓ, and $h_{virt}^{\left(\ell \right)}$ is the virtual node's representation at the same layer. After message‐passing, the virtual node embedding is updated by aggregating the current node states across the graph:

$$h_{\mathrm{pool}}^{\left(\ell \right)}=\sum_{\nu \in V}h_{\nu }^{\left(\ell \right)}$$

where $h_{pool}^{\left(\ell \right)}$ is the graph‐level summary at layer ℓ, computed as a permutation‐invariant sum over all atomic representations $h_{v}^{\left(\ell \right)}$. The pooled representation is then transformed through a learnable nonlinear function $MLP^{\left(\ell \right)}$, typically consisting of two linear layers with intermediate activation and normalization, to produce a virtual node embedding as follows:

$$\Delta \;h_{virt}^{\left(\ell +1\right)}=MLP^{\left(\ell \right)}\;(h_{pool}^{\left(\ell \right)}$$

This mechanism enables the virtual node to function as a contextual relay, progressively refining global semantics while retaining memory of prior states. Finally, the virtual node embedding is updated by residual addition:

$$\;h_{virt}^{\left(\ell +1\right)}=h_{virt}^{\left(\ell \right)}+\Delta h_{virt}^{\left(\ell +1\right)}$$

where the virtual node embedding from the previous layer $h_{virt}^{\left(\ell \right)}\;\in R^{d}$ is incremented by the newly computed update $\Delta h_{virt}^{\left(\ell +1\right)}\in R^{d}$.

The JK mechanism was incorporated into architectures to address this limitation by allowing the model to adaptively aggregate information from multiple message‐passing depths, effectively capturing signals at varying neighborhood ranges. Formally, let $H^{\left(0\right)},\;H^{\left(1\right)},\;\in ,\;H^{\left(L\right)}$ denote the sequence of node embeddings obtained at each of the *L* message‐passing layers. JK aggregation builds a unified node representation through feature concatenation:

$$\;H^{\mathrm{JK}}=\;\mathrm{concat}\;\left[H^{\left(0\right)},\;H^{\left(1\right)},\;\in ,\;H^{\left(L\right)}\right]$$

#### 2.3.3 Homoscedastic Uncertainty

A homoscedastic uncertainty weighting approach [43] was employed to balance task contributions during multitask learning by accounting for task‐dependent variance. For each task *t*, a trainable parameter $a_{t}=\log\;\sigma_{t}^{2}$ defined as the logarithm of the task‐dependent variance was introduced to adaptively scale the classification loss as follows:

$$\ell =\frac{1}{T}\sum_{t=1}^{T}\frac{1}{\sum_{i=1}^{N}m_{i.t}}\sum_{i=1}^{N}m_{i.t}\left(e^{-a_{t}}\ell_{i,t}+a_{t}\right)$$

where $\sigma_{t}^{2}$​ represents the homoscedastic uncertainty associated with task *t*, *T* is the number of tasks, and *N* the number of samples, and $\ell_{i,t}$ is the individual loss for sample *i* in task *t*. The exponential term $e^{-a_{t}}$ adaptively downweights tasks with higher uncertainty, while the additive *a_t_* term penalizes overly large uncertainty estimates, preventing trivial solutions.

### 2.4 Tier‐2 Models

Tier‐2 models were developed to predict in vivo Hershberger and uterotrophic assays, integrating molecular and pathway graphs as complementary feature representations.

#### 2.4.1 Pathway Graphs

Undirected pathway graphs were built from AR‐ [13] and ER‐mediated [14] AOPs by integrating hierarchical AOP structure with statistical co‐occurrence patterns derived from ToxCast/Tox21 predictions. Each node represented an individual assay and was annotated with its Tier‐1 logit, i.e., the raw model output for that task. The hierarchical structure of the AOP was encoded by assigning each assay to a rule level (MIE or KE_k_) and by connecting assays within the same biological pathway (AR or ER) when they belonged to the same rule level or to adjacent levels (e.g., MIE→KE1, KE1→KE2). Statistical associations between assays were incorporated as edge attributes. For each pair of assays *a* and *b*, we computed the normalized pointwise mutual information (nPMI) and Yule's Q values from binarized Tier‐1 predictions using task‐specific calibration thresholds. The nPMI between tasks *a* and *b* was given by:

$$nPMI\;\left(a,b\right)=\frac{log\left(\frac{p\left(a,b\right)}{p\left(a\right)\;p\left(b\right)}\right)}{-\log p\left(a,b\right)}\;\in \;\left[-1,\;1\right]$$

Yule's(*a*, *b*) is the joint probability of both tasks being predicted as positive, and *p*(*a*) and *p*(*b*) are their respective marginal probabilities. In parallel, Yule's Q values derived from the odds ratio were computed as follows:

$$OR\;\left(a,b\right)=\frac{TP\cdot TN}{FP\cdot FN},\;Q\;\left(a,b\right)=\frac{OR-1}{OR+1}\;\in \;\left[-1,\;1\right]$$

where TP, TN, FP, and FN denote the counts of true positives, true negatives, false positives, and false negatives for the pair (*a*, *b*). Finally, these association metrics were combined into a 2D edge‐feature vector *e*_*a*,*b*_ =  [*nPMI*_*a*,*b*_,  *Q*_*a*,*b*_].

#### 2.4.2 Architecture

Four multimodal GNNs were developed using MPNN, GAT, GIN, and AttentiveFP message‐passing mechanisms. Each model employed parallel message‐passing arms for the molecular and pathway graphs, enhanced with GraphNorm [41], skip connections, virtual nodes, and JK mechanisms. The outputs of both encoders were projected into a shared latent dimension and integrated through a bidirectional cross‐attention mechanism. Multi‐head attention (MHA) was computed as:

$$\mathrm{MHA}\;\left(Q,K,V\right)=\;\mathrm{Concat}\left(\mathrm{hea}d_{1},\text{…},\mathrm{hea}d_{h}\right)W^{O}$$

$$\mathrm{hea}d_{i}=\;\mathrm{softmax}\;\left(\frac{QW_{i}^{Q}{\left(KW_{i}^{K}\right)}^{T}}{\sqrt{d_{k}}}\right)VW_{i}^{V}$$

where Q, K, and V represent query, key, and value projections of the input node, *d_k_* is the key dimension, and *W^O^*, $W_{i}^{Q}$, $W_{i}^{K}$, and $W_{i}^{V}$ are learned projection matrices. In the molecule‐to‐pathway (M2P) direction, pathway embeddings were updated as:

$$\;P^{\prime }=\;P+\mathrm{MHA}\left(P,\;M,\;M\right))$$

where P denotes the input pathway node embeddings and M the molecular embeddings. Conversely, in the pathway‐to‐molecule (P2M) direction, molecular representations were updated as:

$$\;M^{\prime }=\;M+\mathrm{Attention}\left(M,\;P^{\prime },\;P^{\prime }\right)$$

where P′ are the updated pathway embeddings. Finally, the refined embeddings (P′ and M′) were pooled and concatenated, then passed through a multilayer perceptron to generate in vivo predictions.

### 2.5 Model Training and Evaluation

All models were implemented in PyTorch v.2.5.1 with PyTorch Geometric v.2.6.1 and trained on a graphics processing unit (GPU) NVIDIA TITAN Xp. Training was limited to 2000 epochs, with early stopping triggered when validation loss improved by < 0.01 over a five‐epoch adaptive window. A ReduceLROnPlateau scheduler was employed to reduce the learning rate when validation loss stagnated beyond the defined patience. To increase efficiency, pruning techniques were employed to terminate both underperforming trials and trials displaying unrealistically low initial validation losses, thereby avoiding degenerate or overfitting‐prone optimization paths. Hyperparameter tuning was performed using Optuna v.4.2.0, with 150 trials per architecture to systematically explore the parameter space. Optimal configurations are reported in Supporting Information. Losses were calculated using binary cross‐entropy (BCE) with logits, computed only over valid labels through a binary mask $m_{i.t\;}\in \left\{0,1\right\}$, which indicates whether sample *i* possesses an annotated value for task *t*. This masking strategy ensures that only valid entries contribute to the loss. Embedding vectors generated during training were retained and later projected via t‐distributed stochastic neighbor embedding (t‐SNE) and kernel density estimation (KDE) to evaluate the structuring of the latent space across epochs.

### 2.6 Statistical Analysis

Model performance was assessed using accuracy (ACC), false positive rate (FPR), false negative rate (FNR), recall, specificity (SP), positive predicted value (PPV), negative predicted value (NPV), geometric (G)‐mean, and area under the receiver operating characteristic (AUROC). All metrics are reported as the mean ± standard deviation (SD) across five independent data splits.

### 2.7 Probabilistic Calibration

Probabilistic calibration was applied in class‐imbalanced settings using a threshold‐moving strategy [44] to adjust the decision boundaries based on raw predicted probabilities. Thresholds were evaluated individually for each task across a 0.0–1.0 range with 0.01 increments on the training and validation sets, and optimal values were defined by the highest G‐mean as follows:

$$\;\tau_{t}^{*}=\underset{\tau \in \left\{0.00,0.01,\;\text{…},1.00\right\}}{\arg max}\sqrt{recall_{t}\left(\tau \right)\cdot \;SP_{t}\left(\tau \right)},\;\;{\hat{y}}_{i,t}\;\left(\tau \right)=1\left[{\hat{p}}_{i,t}\;\geq \;\tau \right]$$

where *recall_t_* (τ) and *SP_t_* (τ) denote recall and specificity, computed at threshold τ for task *t*. The indicator function $1[{\hat{p}}_{i,t}\;\geq \;\tau ]$ assigns a binary label $\;{\hat{y}}_{i,t}\left(\tau \right)$ to each prediction ${\hat{p}}_{i,t}$, with outcome 1 if the predicted probability is greater than or equal to τ, and 0 otherwise. The calibrated thresholds $\tau_{t}^{*}$ were subsequently applied to the test set to reclassify predictions, enhancing the trade‐off between recall and SP while maintaining AUROC.

### 2.8 Cross‐Attentive Explainability

Cross‐attentive explanations were generated by extracting bidirectional attention matrices between the molecular and pathway graphs during inference, using M2P and P2M attention weights to compute node‐level importance scores. Formally, the importance score for a source node *i* was calculated as:

$$h_{i}=\frac{1}{\left|T\left(i\right)\right|}\sum_{j\in T\left(i\right)}A_{\mathrm{ij}}$$

where *A_ij_* denotes the cross‐attention weight from node *i* to target *j*, and *T*(*i*) is the set of valid attended targets. In the M2P direction, *i* corresponds to a molecular node (atom) and *j* to a pathway node (assay). Conversely, in the P2M direction, *i* denotes a pathway node and *j* a molecular node. Interpretability was enhanced by retaining only the top “*k*” attention links between *i* and *j*. Finally, both graphs were rendered in the same spatial frame, with bidirectional cross‐attention links superimposed to highlight the most influential molecular–assay interactions.

### 2.9 Counterfactual Explainability

Stochastic perturbations were applied to the molecular and pathway graphs using rule‐based isosteric and valence‐preserving substitutions, and quasi‐random assay perturbations derived from low‐discrepancy sequences, respectively. Subsequently, the importance score of each node *i* was calculated as follows:

$$\;h_{i}=\frac{1}{K}\sum_{k=1}^{K}\left(p_{0}-p_{i}^{\left(k\right)}\right)$$

where *p*_0_ is the predicted probability for the unperturbed graph, $p_{i}^{\left(k\right)}$ is the predicted probability after *k*‐th perturbation of node *i*, and *K* is the number of perturbation samples. Graph‐based clustering was then performed using connected components to group structurally connected atoms with similar contribution signals, with intra‐cluster enhancement applied as:

$$C_{i}=\mu_{c}+\gamma \left(h_{i}-\mu_{c}\right)$$

where *C_i_* is the cluster‐enhanced contribution score for atom *i*, 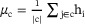 is the mean contribution *c*, and γ > 0 is the cluster‐enhancement factor. Laplacian smoothing was subsequently applied within each cluster to reduce noise while preserving local structure as follows:

$${\tilde{h}}_{i}=\alpha C_{i}+\left(1-\alpha \right)\sum_{j\in N\left(i\right)}\frac{1}{d_{i}}C_{j}$$

where *C_j_* is the cluster‐enhanced score of each neighbor $j\in N\left(i\right)$, whereas α∈[0,1]​ balances self‐vs‐neighbor contributions, *N*(*i*) is the set of neighbors of atom *i*, and *d_i_* =  |*N*(*i*)| its degree. Finally, the ${\tilde{h}}_{i}$ values are normalized (zero mean, unit variance) and projected onto 2D graph coordinates for visualization.

### 2.10 Benchmarking and Ablation Studies

Random Forest (RF) and Support Vector Machine (SVM) models were developed using Tier‐1 logits and/or extended‐connectivity fingerprints with diameter 4 (ECFP4) [45]. These baselines were evaluated using a 5‐fold cross‐validation scheme, with the same test partitions as for the Tier‐2 models to ensure a consistent comparison framework. Then, the held‐out test sets were also evaluated using the U.S. EPA Collaborative Modeling Project for Androgen Receptor Activity (CoMPARA) [46] and Collaborative Estrogen Receptor Activity Prediction Project (CERAPP) [20] consensus models implemented in the OPERA software [47] to provide regulatory‐grade benchmark references. Furthermore, ablation studies were further conducted to quantify the individual contributions of Tier‐1 predictions, molecular graphs, and pathway graphs to the overall predictive performance. Pairwise differences between selected model outputs were verified using two‐sample *t*‐tests computed in GraphPad Prism version 8.0.2 (GraphPad Software, San Diego, CA).

## 3 Results and Discussion

### 3.1 Tier‐1

**FIGURE 1:**
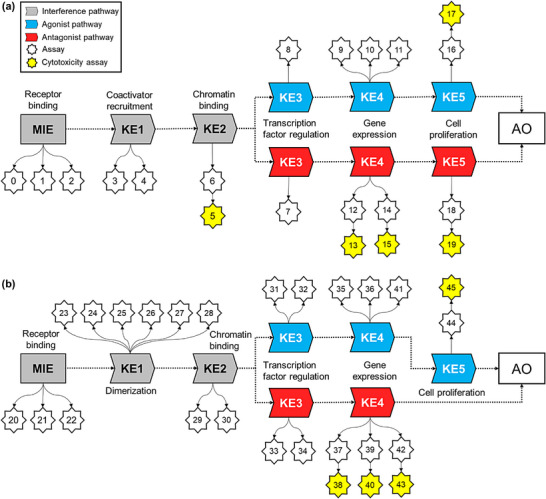
Graphical representation of the (a) AR‐ and (b) ER‐mediated AOPs based on Tox21/ToxCast assays.

Tier‐1 modeling was designed to predict in vitro responses across AR‐ [13] and ER‐mediated [14] assays, establishing the mechanistic foundation for subsequent in vivo predictions. This strategy reflects a central assumption of the AOP framework: perturbations at the MIE level can propagate through the downstream cascade of KEs (MIE→KE_1_→KE_2_→KE_3_→KE_4_→KE_5_), which ultimately culminate in an adverse outcome (AO) at the organism level. Initially, we compiled a dataset of 7,956 chemicals from the U.S. EPA ToxCast/Tox21 program (invitroDB v4.1), covering 46 in vitro assays selected and organized according to the hierarchical structure of AR‐ [13] and ER‐mediated [14] AOPs (Figure 1).

As shown in Figure 1a, 20 in vitro assays were prioritized to capture key mechanistic checkpoints along the androgen signaling axis. This panel comprised three AR‐binding assays (MIE), two co‐regulator recruitment assays (KE1), one chromatin‐binding assay (KE2), two transcription‐factor regulation assays (KE3), five gene‐expression assays (KE4), two cell‐proliferation assays (KE5), and five cytotoxicity assays. Similarly, the estrogen signaling axis (Figure 1b) was represented by 26 in vitro assays that mirror its hierarchical organization. This panel included three receptor‐binding assays (MIE), six dimerization and co‐regulator recruitment assays (KE1), two chromatin‐binding assays (KE2), four transcription‐factor regulation assays (KE3), six gene‐expression assays (KE4), one cell‐proliferation assay (KE5), and four cytotoxicity assays. In both AOPs, cytotoxicity assays were incorporated to provide critical biological context for interpreting agonistic and antagonistic signatures, helping to distinguish true receptor‐mediated pathway perturbations from nonspecific effects driven by generalized cell stress or loss of viability [48, 49]. In contrast, neither the AR‐ nor ER‐related assay panels incorporate metabolic competence or toxicokinetic‐related processes. As a result, assay‐derived AOP cascades may not capture mechanisms that require metabolic activation or detoxification, which are known to alter endocrine disruption in vivo.

**FIGURE 2:**
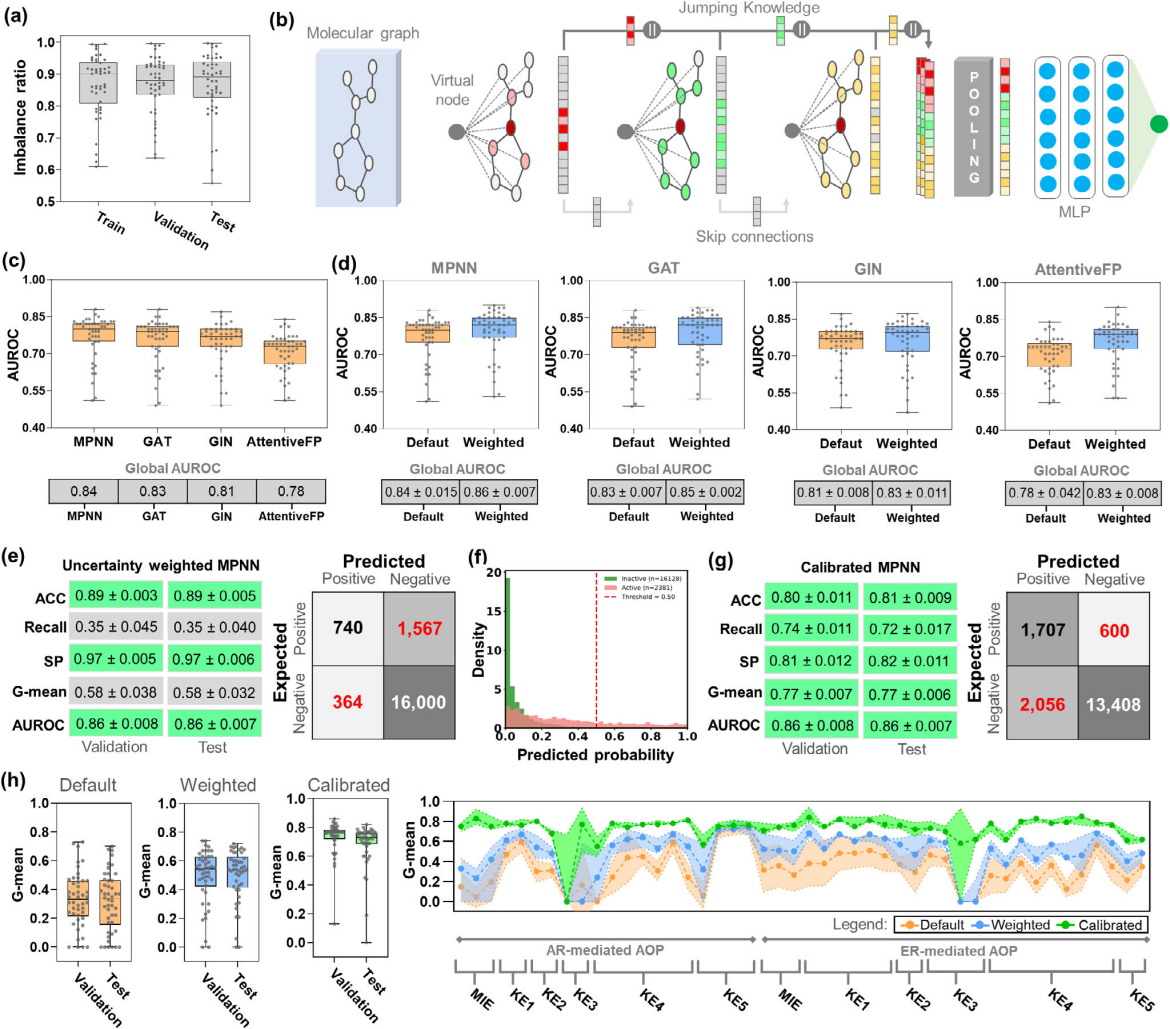
Statistical characteristics of multitask GNNs for predicting outcomes of in vitro AR‐ and ER‐mediated assays. (a) Imbalance ratios across training, validation, and test splits. (b) Schematic representation of multitask GNNs with virtual node, skip connections, and Jumping Knowledge mechanisms. (c) Global AUROC values of four GNN backbones. (d) Comparison of default vs. uncertainty‐weighted loss across GNNs. (e) Global performance metrics of the uncertainty‐weighted MPNN and corresponding confusion matrix computed on the test set. (f) Predicted probability distributions showing class imbalance and suboptimality of the 0.5 threshold. (g) Global performance metrics of the calibrated MPNN model and the corresponding confusion matrix computed on the test set. (h) Task‐level G‐mean values for default, uncertainty‐weighted, and calibrated models across the AR‐ and ER‐mediated AOPs, highlighting the performance gains achieved through the multi‐step optimization pipeline. Individual points and shaded bands represent task‐level means over five independent data splits; metrics are reported as mean ± SD.

MIEs and KEs were subsequently used to train multitask learning models to predict AR‐ and ER‐mediated responses. Modeling this dataset poses a substantial challenge, as the imbalance ratio remained consistently high across training (0.866 ± 0.095), validation (0.868 ± 0.089), and test (0.867 ± 0.099) sets, highlighting the predominance of inactives (Figure 2a). To address this, four multitask GNN architectures were explored (MPNN, GAT, GIN, and AttentiveFP), each optimized with virtual node embeddings, skip connections, and JK mechanisms to improve chemical semantic representation and mitigate oversmoothing and oversquashing during training (Figure 2b). Performance evaluation revealed global AUROC values of 0.84 ± 0.015 for MPNN, 0.83 ± 0.007 for GAT, 0.81 ± 0.008 for GIN, and 0.78 ± 0.042 for AttentiveFP, identifying the multitask MPNN as the most effective backbone for Tier‐1 predictions (Figure 2c). Detailed statistical characteristics for all multitask GNN models are provided in Table S4.

To further address heterogeneity across tasks, we implemented a homoscedastic uncertainty weighting approach [43] to adaptively balance loss contributions during multitask learning. In this approach, each task is assigned a trainable variance parameter that downweights highly uncertain tasks, preventing them from dominating the optimization process. Meanwhile, a regularization term penalizes inflated variance estimates to avoid trivial solutions. As shown in Figure 2d, introducing uncertainty weighting consistently improved performance across all GNN backbones. For the MPNN, the global AUROC increased from 0.84 ± 0.015 to 0.86 ± 0.007 (+2.4%), reinforcing its role as the strongest baseline architecture. The GAT model exhibited a similar gain, rising from 0.83 ± 0.007 to 0.85 ± 0.002 (+2.4%), while the GIN backbone improved from 0.81 ± 0.008 to 0.83 ± 0.011 (+2.5%). AttentiveFP showed the most significant relative increase, from 0.78 ± 0.042 to 0.83 ± 0.008 (+6.4%), reflecting a substantial benefit from task‐dependent uncertainty modulation.

As shown in Figure 2e, the uncertainty‐weighted MPNN achieved high overall ACC on the test set (0.89 ± 0.005), reflecting its strong ability to classify the dominant inactive class. Nevertheless, the severe class imbalance resulted in an asymmetric error profile: SP remained high (0.97 ± 0.006), whereas recall was substantially lower (0.35 ± 0.040), indicating that a significant fraction of actives was systematically misclassified. This imbalance was further captured by the G‐mean, which remained modest (0.58 ± 0.032), despite the high discriminative ability indicated by the AUROC (0.86 ± 0.007). The distribution of predicted probabilities (Figure 2f) further illustrates this bias, with inactives clustering near zero and most actives falling below the standard 0.5 decision boundary, highlighting that this default threshold is not optimal in such an imbalanced setting.

Probabilistic calibration [44] was then applied to the best‐performing model (MPNN) using task‐specific thresholds optimized by G‐mean. This calibration effectively shifts the decision boundaries on a task‐by‐task basis, moving them away from the default 0.5 threshold toward values that balance recall and SP. As shown in Figure 2g, this strategy markedly increased recall (0.72 ± 0.017) while maintaining balanced SP (0.82 ± 0.011). Consequently, the test‐set G‐mean increased from 0.58 ± 0.032 to 0.77 ± 0.006, representing an approximately 33% relative improvement. At the individual‐assay level (Figure 2h), the multi‐step optimization pipeline, comprising the default models, uncertainty weighting, and subsequent task‐specific threshold calibration, yielded systematic improvements in G‐mean across most AR‐ and ER‐mediated endpoints. Calibration consistently improved performance stability across tasks and increased their corresponding G‐mean values, particularly for assays most affected by severe class imbalance. Only a small subset of endpoints showed minimal improvement, reflecting intrinsic limitations imposed by the extreme sparsity of active samples and the high imbalance ratios in those tasks.

### 3.2 Tier‐2

**FIGURE 3:**
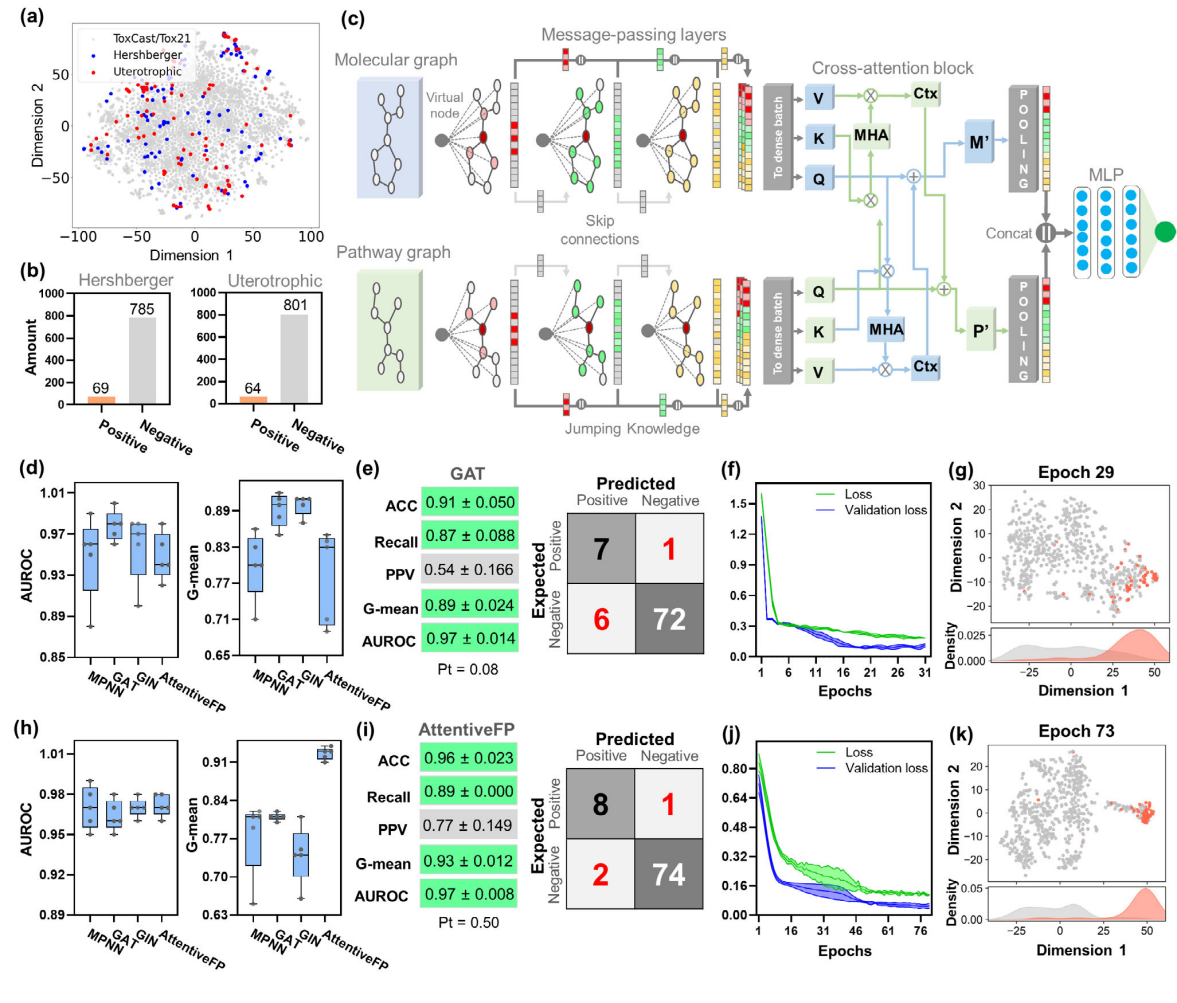
Statistical characteristics of multimodal GNN framework for predicting outcomes in Hershberger and uterotrophic assays. (a) t‐SNE projection of the original Hershberger and uterotrophic datasets onto the ToxCast/Tox21 chemical space. (b) Distribution of positive and negative samples in the Hershberger and uterotrophic datasets after data augmentation. (c) Schematic representation of the overall multimodal GNN architecture. (d) Test set AUROC and G‐mean performances of multimodal GNN backbones (MPNN, GAT, GIN, AttentiveFP) for the Hershberger assay. (e) Performance metrics and confusion matrix of the best Hershberger model (GAT‐based). (f) Training and validation loss curves for the GAT Hershberger model. (g) t‐SNE projection of Hershberger predictions at the optimal epoch (epoch 29), illustrating separation of active and inactive compounds together with corresponding KDE density profiles. (h) Test set AUROC and G‐mean performances of multimodal GNN backbones for the uterotrophic assay. (i) Performance metrics and confusion matrix of the best uterotrophic model (AttentiveFP‐based). (j) Training and validation loss curves for the AttentiveFP uterotrophic model. (k) t‐SNE projection of uterotrophic predictions at the optimal epoch (epoch 73), with corresponding KDE density distributions. Individual points in boxplots represent values from five independent test splits; metrics are reported as mean ± SD.

Tier‐2 modeling was designed to predict in vivo endocrine outcomes, specifically the Hershberger and uterotrophic assays, by integrating chemical features with pathway‐derived logits from the Tier‐1 model. The Hershberger dataset [33] comprised 136 compounds, with activity defined by androgen‐dependent changes in the weights of accessory sex tissues. The uterotrophic dataset [34] comprised 118 compounds, where estrogenic activity was determined by measuring uterine weight changes in immature or ovariectomized female rodents. Notably, compiled in vivo data provided broad coverage of the ToxCast/Tox21 chemical space (Figure 3a), indicating that the Tier‐2 sets are broadly aligned with the chemical diversity covered by these assays.

To expand the limited sample size of both assays, we applied a structured data augmentation strategy that incorporated putative inactive compounds in both datasets. The data augmentation expanded the Hershberger dataset by adding 717 compounds and increased the uterotrophic dataset to a total of 747 compounds. Approximately 82% of the augmented entries met the criteria for hard negatives, defined as compounds exhibiting activity in parallel biological pathways or unrelated assay endpoints while remaining consistently inactive across AR‐ and ER‐mediated MIE and KE5 assays. As shown in Figure 3b, the Hershberger dataset consisted of 69 positive and 785 negative samples (imbalance ratio = 0.92), while the uterotrophic dataset comprised 64 positive and 801 negative samples (imbalance ratio = 0.93).

Subsequently, a mechanistic context was incorporated by generating pathway graphs derived from the AR‐ and ER‐mediated AOPs, in which nodes represent individual Tier‐1 assays and edges encode their hierarchical relationships and co‐occurrence patterns. These pathway graphs were integrated with molecular graphs through a multimodal GNN architecture (Figure 3c), where message‐passing layers (MPNN, GAT, GIN, or AttentiveFP) processed each graph separately, and bidirectional cross‐attention blocks enabled information exchange between them. The refined molecular and pathway embeddings were pooled and concatenated, yielding optimized representations for predicting in vivo outcomes.

All multimodal GNN architectures demonstrated strong predictive performance for the Hershberger assay, with test set AUROC values consistently above 0.95 (Figure 3d). Detailed statistical outputs for all models are provided in Table S5. Among them, the calibrated GAT‐based model (probability threshold = 0.08 ± 0.075) yielded the best test set AUROC (0.97 ± 0.014). This model also reached the highest ACC (0.91 ± 0.050), together with the highest ACC (0.91 ± 0.050) and a favorable balance between recall (0.87 ± 0.088) and specificity (0.91 ± 0.062). These metrics reflect a G‐mean of 0.89 ± 0.024, consistent with the behavior observed in the associated confusion matrix (Figure 3e). Training dynamics supported the robustness of this model (Figure 3f), with both the training and validation losses converging stably across the five seeded data splits. In addition, the t‐SNE projection at the optimal epoch (Figure 3g) revealed a clear separation between positives (orange) and negatives (gray). The corresponding KDE distributions confirmed minimal overlap between the two classes, highlighting the model's ability to generate well‐structured latent representations that discriminate against AR‐mediated responses.

Multimodal GNNs also achieved strong performance in the uterotrophic assay, with all architectures achieving test‐set AUROC values above 0.96 (Figure 3h). Detailed statistical outputs for all models are provided in Table S6. Among them, the uncalibrated AttentiveFP‐based model (probability threshold = 0.50) yielded the best overall results, achieving a test set AUROC of 0.97 ± 0.008. This model maintained high ACC (0.96 ± 0.023) and demonstrated the most balanced trade‐off between recall (0.89 ± 0.030) and SP (0.96 ± 0.026), resulting in a G‐mean of 0.93 ± 0.012 (Figure 3i). Training dynamics further confirmed the robustness of this architecture (Figure 3j), with both the training and validation losses converging smoothly and consistently across the five data splits. The t‐SNE projections of learned embeddings (Figure 3k) revealed distinct clustering of positives (orange) and negatives (gray), with minimal overlap in KDE density, reinforcing the strength of AttentiveFP in modeling ER‐mediated responses.

### 3.3 Benchmarking and Ablation Studies

**FIGURE 4:**
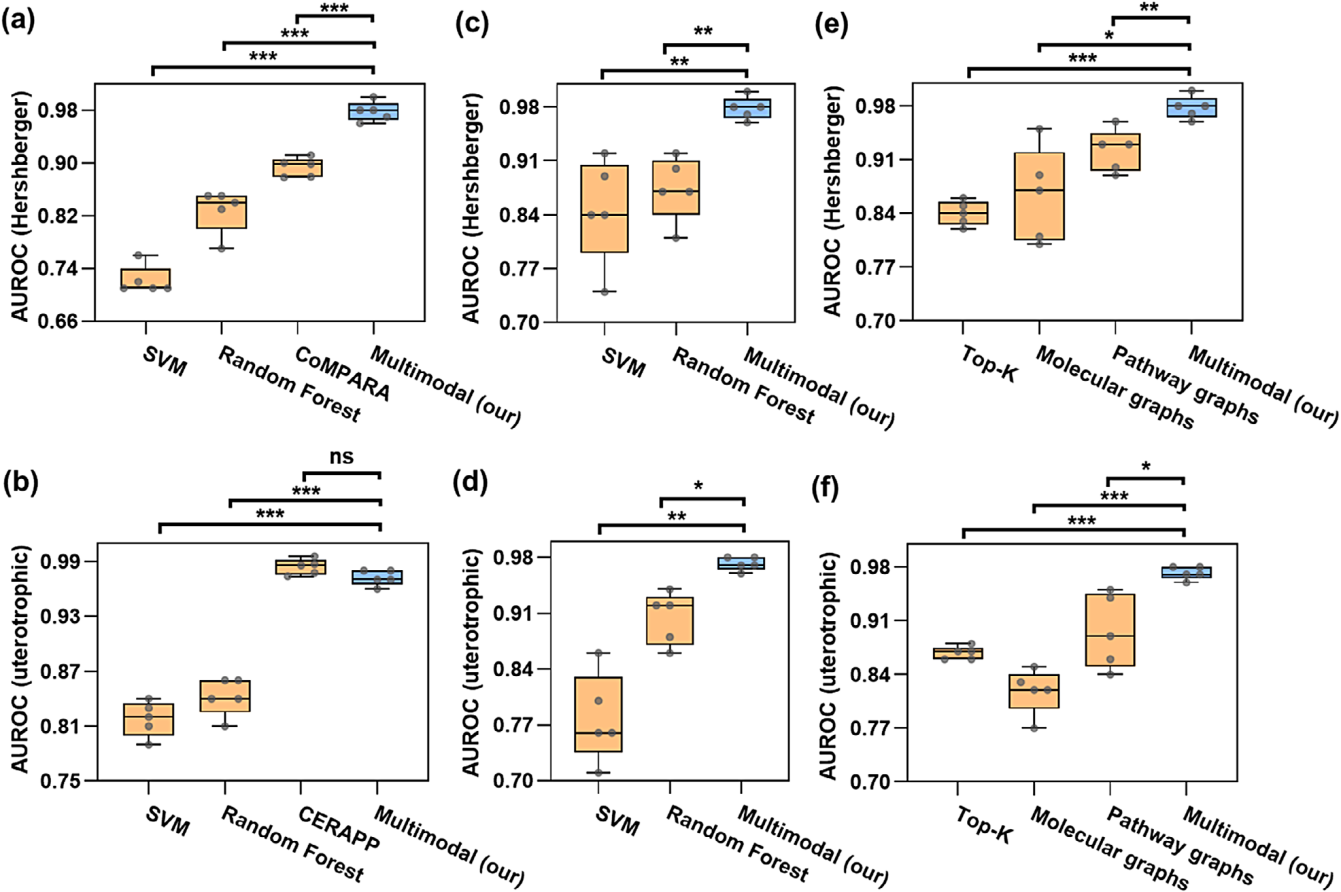
Benchmarking and ablation studies of multimodal GNNs for predicting outcomes in Hershberger and uterotrophic assays. (a,b) AUROC comparison of ECFP4‐based SVM and Random Forest baselines and the U.S. EPA consensus models (CoMPARA, CERAPP) against the proposed multimodal GNNs. (c,d) AUROC comparison of SVM and Random Forest baselines generated from ECFP4 fingerprints and Tier‐1 logits against the proposed multimodal GNNs. (e‐f) AUROC comparison of the proposed multimodal GNNs with the top‐k AOP‐level algorithm and ablated GNN architectures. Individual points in boxplots represent values from five independent test splits; ns = not significant difference; ^*^ *p* < 0.05; ^**^ *p* < 0.01; ^***^ *p* < 0.001 (two‐sample t‐tests).

We benchmarked the proposed Tier‐2 multimodal models against standard machine learning baselines (SVM and Random Forest using ECFP4) and U.S. EPA reference models (CoMPARA [46] and CERAPP) [20]. As shown in Figure 4a, the multimodal GAT model developed for the Hershberger assay achieved substantially higher AUROC values compared with SVM (0.72 ± 0.021) and Random Forest (0.83 ± 0.033), with all pairwise differences reaching strong statistical significance (two‐sample *t*‐tests, *p* < 0.001). The multimodal model also outperformed the CoMPARA consensus model (0.89 ± 0.014), representing a highly significant improvement (*p* < 0.001). In the uterotrophic baseline (Figure 4b), our multimodal AttentiveFP model also outperformed both SVM (0.82 ± 0.019) and Random Forest (0.84 ± 0.020), again with strong statistical support (*p* < 0.001). In contrast, the comparison with CERAPP (0.98 ± 0.008; *p* > 0.05) showed no statistically significant difference, indicating that the two models perform equivalently.

We then assessed the predictive value of simpler input representations by evaluating SVM and Random Forest models combining ECFP4 fingerprints and Tier‐1 logits. In the Hershberger assay (Figure 4c), the SVM baseline achieved an AUROC of 0.84 ± 0.068, representing a significant improvement over its ECFP4‐only counterpart (+16.7%). The Random Forest model reached 0.87 ± 0.041, which corresponds to a small reduction relative to its counterpart (−1.2%). Both approaches, however, remained significantly below the multimodal GAT model (*p* < 0.01). A similar pattern was observed in the uterotrophic assay. As shown in Figure 4d, the SVM model (AUROC = 0.78 ± 0.055) showed a small decrease compared with its ECFP4‐only version (−4.9%), whereas the Random Forest model improved slightly (0.90 ± 0.032; +7.1%). Nonetheless, both models underperformed relative to the multimodal AttentiveFP model (*p* < 0.01 and < 0.05, respectively).

Subsequently, an ablation analysis was conducted to assess the contribution of each information source (Tier‐1 model, molecular graphs, and pathway graphs) to the overall predictive performance of the Tier‐2 models. Initially, the top‐*k* method (Algorithm 1) was developed to evaluate whether the extent of perturbation across MIEs and KEs could serve as a proxy for in vivo responses. This approach classifies a compound as positive when the number of activated levels (MIEs and KEs) within the AOP exceeds a predefined threshold, reflecting the principle that progression across multiple key events increases the likelihood of an adverse outcome [50, 51].

ALGORITHM 1 **Top‐*k* algorithm for in vivo endocrine disruption classification**

**Table:** 

1:	**Input**: Tier‐1 predictions *P*, thresholds τ
2:	**Initialize** selected tasks *T*
3:	**while** session active **do**
4:	**Binarize** *B* = [*P* ≥ τ]
5:	**Apply** viability filter → *B*′
6:	**Identify** effective tasks *E*
7:	**Count** active levels $L=|\left\{\ell \in \mathrm{levels}|\exists_{j}\in E:{B^{\prime }}_{:,j}=1\right\}|$
8:	**if** 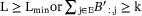 **then**
9:	**Assign** toxic
10:	**else** non‐toxic

As shown in Figure 4e, the top‐*k* classifier for the Hershberger top‐*k* predictor achieved an AUROC of 0.84 ± 0.015 (−13.4%), which was significantly lower than the multimodal GAT model (*p* < 0.001). A similar pattern emerged for the uterotrophic top‐k predictor (Figure 4f), which achieved an AUROC of 0.86 ± 0.008 (−11.3%), significantly lower than the multimodal AttentiveFP model (*p* < 0.001). These findings indicate that aggregated perturbations across MIEs and KEs capture meaningful AOP‐level progression. However, this rule‐based representation lacks the pathway‐level relational structure needed to match the predictive performance of our multimodal framework.

Ablation of individual components of multimodal architecture revealed distinct contributions from the molecular and pathway representations. In the Hershberger assay (Figure 4e), the molecular‐only model achieved an AUROC of 0.86 ± 0.065 (−12.1%), while the pathway‐only model reached 0.92 ± 0.027 (−5.4%). However, both ablations remained significantly below the multimodal GAT model (*p* < 0.05 and < 0.01, respectively). In the uterotrophic assay (Figure 4f), the molecular‐only model reached an AUROC of 0.81 ± 0.029 (−16.5%), whereas the pathway‐only model achieved an AUROC of 0.89 ± 0.048 (−8.2%). Both ablations again underperformed relative to the multimodal AttentiveFP model (*p* < 0.001 and *p* < 0.05). Overall, each modality provides meaningful information, but neither achieves the predictive performance of the multimodal framework, underscoring the need for molecular–pathway fusion for optimal Tier‐2 prediction.

## 4 Retrospective Analysis with Literature Compounds

To further interrogate the robustness and translational value of the Tier‐2 models, a retrospective analysis was performed using compounds identified through a systematic literature review. Equivocal or conflicting reports, as well as mixtures and compounds associated with systemic effects such as changes in body weight, were excluded from the analysis. This evaluation included 18 chemicals with androgen‐related outcomes directly relevant to the Hershberger assay and 26 chemicals with estrogen‐related outcomes relevant to the uterotrophic assay (Tables 1 and 2). The selected compounds encompassed both substances tested in OECD guideline studies and others with well‐documented in vivo outcomes strongly indicative of the same endocrine endpoints, thereby providing a stringent and diverse benchmark for model assessment.

**TABLE 1:** Retrospective analysis of the Hershberger model with chemicals identified from the literature.

Compound (CAS)	usage	Structure	Predicted outcome (probability)	CoMPARA (Probability)			Expected outcome	Adverse outcome (in vivo)	Refs.
				Agonist	Antagonist	Binding			
Homosalate (118‐56‐9)	UV filter	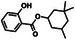	Positive (0.49)	Inactive (0.03)	Inactive (0.33)	Inactive (0.38)	Negative	No alterations in androgen‐dependent tissues (up to the limit dose of 1000 mg/kg/day)	U.S. EPA, reference number: N135‐249 [52]
Padimate‐O (21245‐02‐3)	UV filter	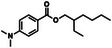	Positive (0.12)	Inactive (0.32)	Inactive (0.31)	Inactive (0.056)	Positive	Decrease in glans penis, bulbocavernous muscle complex, and seminal vesicle weights in castrated rats (1000 mg/kg/day, 10 days, po)	U.S. EPA, reference number: N135‐249 [52]
Ensulizole (27503‐81‐7)	UV filter	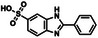	Negative (0.004)	Inactive (0.14)	Active (0.61)	Active (0.73)	Negative	No alterations in androgen‐dependent tissues (up to the limit dose of 1000 mg/kg/day)	U.S. EPA, reference number: N135‐248 [53]
Avobenzone (70356‐09‐1)	UV filter	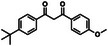	Positive (0.20)	Inactive (0.05)	Active (0.79)	Active (0.77)	Negative	No alterations in androgen‐dependent tissues (up to the limit dose of 1000 mg/kg/day)	U.S. EPA, reference number: N135‐248 [53]
Miltefosine (58066‐85‐6)	Antileishmanial drug	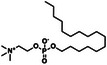	Positive (0.14)	Inactive (0.26)	Inactive (0.31)	Inactive (0.33)	Positive	Atrophy of prostate and seminal vesicles in rats (3.16–6.19 mg/kg/day, 52 weeks)	FDA, application number: 204684Orig1s000 [54]
Oxyfluorfen (42874‐03‐3)	Herbicide	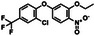	Positive (0.87)	Inactive (0.00)	Active (0.57)	Active (0.52)	Positive	Reduced prostate/seminal vesicle weight in castrated rats (62.5 mg/kg/day, 10 days, po)	Murr et al. [55]
Roflumilast (162401‐32‐3)	Anti‐inflammatory drug	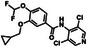	Positive (0.82)	Inactive (0.03)	Active (0.82)	Active (0.73)	Positive	Atrophy of prostate and seminal vesicles in rats (0.8 mg/kg/day)	FDA, application number: 022522orig1s000 [56]
Enzalutamide (915087‐33‐1)	Androgen antagonist drug	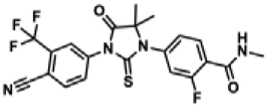	Positive (0.49)	Inactive (0.32)	Active (0.66)	Active (0.70)	Positive	Atrophy of prostate and seminal vesicles in rats (30 mg/kg/day, 26 weeks)	FDA, application number: 203415Orig1s000 [57]
Obeticholic acid (459789‐99‐2)	Bile acid drug	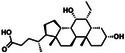	Positive (0.98)	Active (0.66)	Active (0.54)	Active (0.67)	Positive	Reduced testes/epididymides size and prostate atrophy in rats (100–200 mg/kg/day)	FDA, application number: 207999Orig1s000 [58]
O‐tolidine (119‐93‐7)	Intermediate for dye production	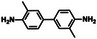	Positive (0.44)	Inactive (0.00)	Active (0.95)	Active (0.92)	Positive	Seminal vesicle hypertrophy in immature rats (10–20 mg/kg/day, 7 days, ip)	Shangguan et al. [59]
N‐phenyl‐2‐naphthylamine (135‐88‐6)	Rubber antioxidant	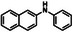	Negative (0.002)	Inactive (0.00)	Active (0.61)	Active (0.76)	Positive	Seminal vesicle hypertrophy in immature rats (5–20 mg/kg/day, 7 days, ip)	Shangguan et al. [59]
Cylindrospermopsin (143545‐90‐8)	Toxin produced by freshwater cyanobacteria	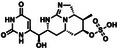	Negative (0.002)	Inactive (0.33)	Inactive (0.03)	Inactive (0.07)	Negative	No alterations in androgen‐dependent tissues in castrated rats (22.5–90 µg/kg/day)	Casas‐Rodríguez et al. [60]
Microcystin‐LR (101043‐37‐2)	Toxin produced by freshwater cyanobacteria	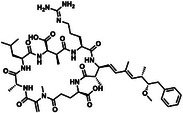	Negative (0.04)	Active (0.54)	Active (0.57)	Active (0.51)	Negative	No alterations in androgen‐dependent tissues in castrated rats (22.5–90 µg/kg/day)	Casas‐Rodríguez et al. [60]
Linalool (78‐70‐6)	Essential oil	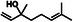	Negative (0.003)	Inactive (0.00)	Inactive (0.00)	Inactive (0.00)	Negative	No alterations in androgen‐dependent tissues (100–1000 µg/kg/day)	Hareng et al. [61]
7α‐methyltestosterone (7642‐58‐2)	Androgen agonist candidate	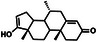	Positive (0.98)	Active (0.67)	Active (0.54)	Active (0.70)	Positive	Increase in ventral prostate and seminal vesicle weights in rats (0.7–11.2 µmol/rat, 7 days, sc)	Lee et al. [62]
7α‐ethyltestosterone	Androgen agonist candidate	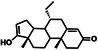	Positive (0.81)	Active (0.69)	Active (0.58)	Active (0.69)	Positive	Increase in ventral prostate and seminal vesicle weights in rats (0.7–11.2 µmol/rat, 7 days, sc)	Lee et al. [62]
acetyl triethyl citrate (77‐89‐4)	Plasticizer of cellulose resin	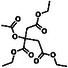	Negative (0.002)	Inactive (0.00)	Inactive (0.00)	Inactive (0.00)	Negative	No alterations in androgen‐dependent tissues (20–500 µg/kg/day)	Sung et al. [63]
acetyl tributyl citrate (77‐90‐7)	Plasticizer found in nail polish	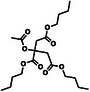	Negative (0.006)	Inactive (0.30)	Inactive (0.02)	Inactive (0.38)	Negative	No alterations in androgen‐dependent tissues (20–500 µg/kg/day)	Sung et al. [63]

**TABLE 2:** Retrospective analysis of the uterotrophic model with chemicals identified from the literature.

Compound (CAS)	Usage	Structure	Predicted outcome (probability)	CERAPP (probability)			Expected outcome	Adverse outcome (in vivo)	Refs.
				Agonist	Antagonist	Binding			
1,1,1‐tris(4‐hydroxyphenyl)ethane (27955‐94‐8)	Crosslinking agent for polymer synthesis	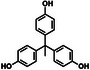	Positive (0.88)	Active (1.00)	Active (1.00)	Active (1.00)	Positive	Increased uterine weight in mice (300 mg/kg/day, 7 days)	Igarashi et al. [64]
4,4′‐butylidenebis[6‐tert‐butyl‐3‐methylphenol] (85‐60‐9)	Antioxidant for polyolefins and rubber	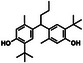	Positive (0.83)	Active (0.82)	Active (0.81)	Active (0.90)	Positive	Decreased uterine weight in mice (100 mg/kg/day, 7 days)	Igarashi et al. [64]
2‐isopropoxy‐2‐phenylacetophenone (6652‐28‐4)	Photoinitiator for UV‐curable resins and coatings	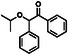	Negative (0.004)	Inactive (0.36)	Active (0.69)	Active (0.75)	Negative	No uterine weight alterations were observed at 30–1000 mg/kg/day over 7 days	Igarashi et al. [64]
Trimethylolpropane trimethacrylate (3290‐92‐4)	Crosslinking agent for polymer synthesis	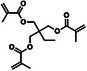	Negative (0.005)	Inactive (0.00)	Inactive (0.00)	Inactive (0.02)	Negative	No uterine weight alterations were observed at 30–300 mg/kg/day over 7 days	Igarashi et al. [64]
Triphenyl phosphate (115‐86‐6)	Flame retardant	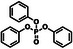	Negative (0.001)	Inactive (0.23)	Active (0.75)	Active (0.82)	Negative	No uterine weight alterations were observed at 30–1000 mg/kg/day over 7 days	Igarashi et al. [64]
2,4‐Di‐tert‐butylphenol (96‐76‐4)	UV stabilizer intermediate	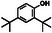	Negative (0.004)	Inactive (0.20)	Active (0.79)	Active (0.78)	Negative	No uterine weight alterations were observed at 30–300 mg/kg/day over 7 days	Igarashi et al. [64]
Dibenzoylmethane (120‐46‐7)	Vinyl chloride stabilizer	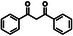	Negative (0.003)	Active (0.58)	Inactive (0.14)	Active (0.86)	Positive	Uterine weight reduction in mice (30 mg/kg/day, 7 days, subcutaneous)	Igarashi et al. [64]
Tricresyl phosphate (78‐32‐0)	Flame retardant	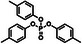	Negative (0.12)	Inactive (0.10)	Active (0.79)	Active (0.83)	Positive	Uterine weight reduction in mice (30 mg/kg/day, 7 days, subcutaneous)	Igarashi et al. [64]
Triphenylsilanol (791‐31‐1)	Silicone polymer intermediate	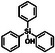	Negative (0.01)	Active (0.54)	Active (0.64)	Active (0.81)	Positive	Uterine weight reduction in mice (30 mg/kg/day, 7 days, subcutaneous)	Igarashi et al. [64]
2‐ethylhexyl diphenyl phosphite (15647‐08‐2)	Antioxidant for plastics and rubber	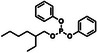	Negative (0.001)	Inactive (0.35)	Inactive (0.33)	Active (0.79)	Negative	No uterine weight alterations were observed at 15–1000 mg/kg/day over 7 days	Igarashi et al. [64]
Linalool (78‐70‐6)	Essential oil	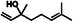	Negative (0.005)	Inactive (0.00)	Inactive (0.00)	Inactive (0.03)	Negative	No increase in estrogen‐dependent tissues (100–1000 µg/kg/day)	Hareng et al. [61]
Raloxifene (198481‐32‐2)	Selective estrogen receptor modulator	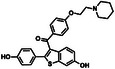	Positive (0.88)	Inactive (0.36)	Inactive (0.12)	Active (0.92)	Positive	Increased uterine weight in immature rats (0.5–5 mg/kg over 3 days)	Komm et al. [65]
Metamizole (68‐89‐3)	Antipyretic drug	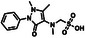	Negative (0.004)	Inactive (0.00)	Inactive (0.00)	Inactive (0.00)	Negative	No uterine weight alterations were observed at 50–200 mg/kg/day over 3 days	Passoni et al. [66]
Tetrazolium violet (1719‐71‐7)	Cell viability dye	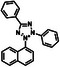	Negative (0.02)	Inactive (0.24)	Active (0.71)	Active (0.54)	Negative	No uterine weight alterations were observed at 30 mg/kg/day over 7 days	Ohta et al. [67]
Physostigmine (57‐64‐7)	Antiglaucoma drug	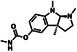	Negative (0.004)	Inactive (0.00)	Inactive (0.00)	Inactive (0.029)	Negative	No uterine weight alterations were observed at 3 mg/kg/day over 7 days	Ohta et al. [67]
o‐Cresolphthalein (596‐27‐0)	pH indicator and reagent for calcium determination	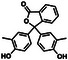	Negative (0.02)	Active (0.91)	Active (0.87)	Active (0.95)	Negative	No uterine weight alterations were observed at 1000 mg/kg/day over 7 days	Ohta et al. [67]
Pigment orange (12236‐62‐3)	Synthetic azo dye for textiles and plastics	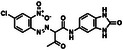	Negative (0.004)	Inactive (0.00)	Inactive (0.33)	Inactive (0.05)	Negative	No uterine weight alterations were observed at 300 mg/kg/day over 7 days	Ohta et al. [67]
2‐[bis(4‐hydroxyphenyl)methyl]benzylalcohol (81‐92‐5)	Synthetic intermediate in dye	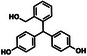	Positive (0.86)	Active (1.00)	Active (1.00)	Active (0.98)	Positive	Increased uterine weight in mice (100 mg/kg/day over 7 days)	Ohta et al. [67]
Neofuchsine (3248‐91‐7)	Biological stain and textile dye	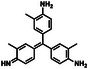	Positive (0.82)	Inactive (0.31)	Active (0.61)	Active (0.50)	Positive	Increased uterine weight in mice (300 mg/kg/day over 7 days)	Ohta et al. [67]
α‐Naphtholbenzein (6948‐88‐5)	pH indicator dye	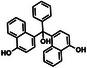	Positive (0.77)	Active (0.62)	Active (0.49)	Active (0.51)	Positive	Increased uterine weight in mice (300 mg/kg/day over 7 days)	Ohta et al. [67]
N,N‐diphenyl‐p‐phenylenediamine (2350‐01‐8)	Antioxidant and stabilizer for rubber and lubricants	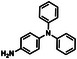	Positive (0.59)	Active (0.50)	Inactive (0.28)	Active (0.65)	Positive	Increased uterine weight in mice (1000 mg/kg/day over 7 days)	Ohta et al. [67]
2,2‐dihydroxy‐4,4’‐dimethoxybenzophenone (131‐54‐4)	UV absorber and stabilizer in plastics	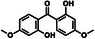	Positive (0.76)	Active (0.85)	Active (0.86)	Active (0.87)	Positive	Increased uterine weight in mice (1000 mg/kg/day over 7 days)	Ohta et al. [67]
Mitotane (53‐19‐0)	Antineoplastic drug	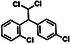	Positive (0.82)	Active (0.85)	Active (0.86)	Active (0.92)	Positive	Case report of a child with uterine enlargement (2.8 g/m^2^/day, 10 months), reversed by anastrozole (1 mg/day, 3 weeks)	Riedmeier et al. [68]
Toremifene (89778‐26‐7)	Selective estrogen receptor modulator	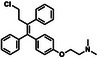	Positive (0.85)	Inactive (0.09)	Inactive (0.06)	Active (1.00)	Positive	Increased uterine weight in rats (1 mg/kg/day subcutaneous, 3 days); partial estrogen agonist	Carthew et al. [69]
Ospemifene (128607‐22‐7)	Selective estrogen receptor modulator	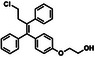	Positive (0.83)	Inactive (0.47)	Active (0.54)	Active (0.61)	Positive	Increased uterine weight in rats (0.3–10 mg/kg/day, 28 days)	Qu et al. [70]
Norethisterone enantate (3836‐23‐5)	Contraceptive drug	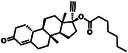	Positive (0.71)	Active (0.86)	Active (0.87)	Active (0.80)	Positive	Increased uterine weight in rats (2–4 mg/kg/day, 10 days)	Srivastava (1981) [71]

Application of the Tier‐2 framework to this independent dataset revealed strong concordance between predicted and observed outcomes. The Hershberger model correctly classified 15 of 18 compounds (88%), while the uterotrophic model achieved the same level of accuracy, correctly identifying 23 of 26 cases (88%). These results align with the performance ranges typically reported for widely used benchmark frameworks. In this dataset, CoMPARA correctly predicted 13 of 18 Hershberger‐relevant compounds (72%), and CERAPP accurately classified 21 of 26 uterotrophic compounds (81%).

Among the compounds evaluated, specific examples illustrate the predictive capacity of the Tier‐2 framework. Padimate‐O (CAS no. 21245‐02‐3), a UV‐B sunscreen filter, was accurately predicted as positive in the Hershberger assay by our multimodal GAT model (probability = 0.12; threshold = 0.08). Consistent with experimental evidence, in vivo studies report decreases in glans penis, bulbocavernous muscle complex, and seminal vesicle weights in rats orally exposed to 1000 mg/kg/day for 10 days [52]. In contrast, ensulizole (CAS no. 27503‐81‐7), another UV filter, was correctly predicted as negative (probability = 0.004; threshold = 0.08), in agreement with Hershberger assays, which showed no alterations in androgen‐dependent tissues up to the limit dose of 1000 mg/kg/day [53].

The application of the Tier‐2 framework to the uterotrophic assay further illustrates the alignment between predicted outcomes and experimental evidence. The compound 4,4′‐butylidenebis[6‐tert‐butyl‐3‐methylphenol] (CAS no. 85‐60‐9), an antioxidant widely used in polyolefins and rubber applications, was accurately predicted as positive in the uterotrophic assay by our multimodal AttentiveFP model (probability = 0.83, threshold = 0.50). Experimental evidence supported this prediction, with in vivo uterotrophic studies reporting a significant decrease in uterine weight in mice following exposure at 100 mg/kg/day for 7 days [64]. In contrast, 2‐ethylhexyl diphenyl phosphite (CAS no. 15647‐08‐2), an antioxidant commonly employed in plastics and rubber manufacturing, was predicted as negative for uterotrophic activity (probability = 0.001, threshold = 0.50). This prediction was consistent with in vivo data, as no alterations in uterine weight were detected in mice exposed to doses ranging from 15 to 1000 mg/kg/day over 7 days [64].

## 5 Tier‐2 Explainability

In addition to assessing the model's performance, it is often beneficial to examine the trained model's “black box” to gain a deeper understanding of which molecular substructures and pathway‐level assays most strongly drive in vivo endocrine disruption. To enable explainability, the present framework incorporates two original strategies designed in this study: cross‐attention mapping and counterfactual perturbations. Cross‐attention analysis was performed bidirectionally, mapping pathway assays to molecular substructures (P2M) and, conversely, linking molecular nodes back to their most influential AOP assays (M2P). This dual perspective provides model‐derived, non‐causal associations between atomic features and AR‐ and ER‐mediated assay nodes, highlighting how chemical motifs relate to pathway‐level perturbation patterns learned by the model. Complementing this mechanistic mapping, counterfactual perturbations were employed to generate alternative scenarios, enabling the evaluation of how molecular modifications or pathway disruptions may alter downstream outcomes.

**FIGURE 5:**
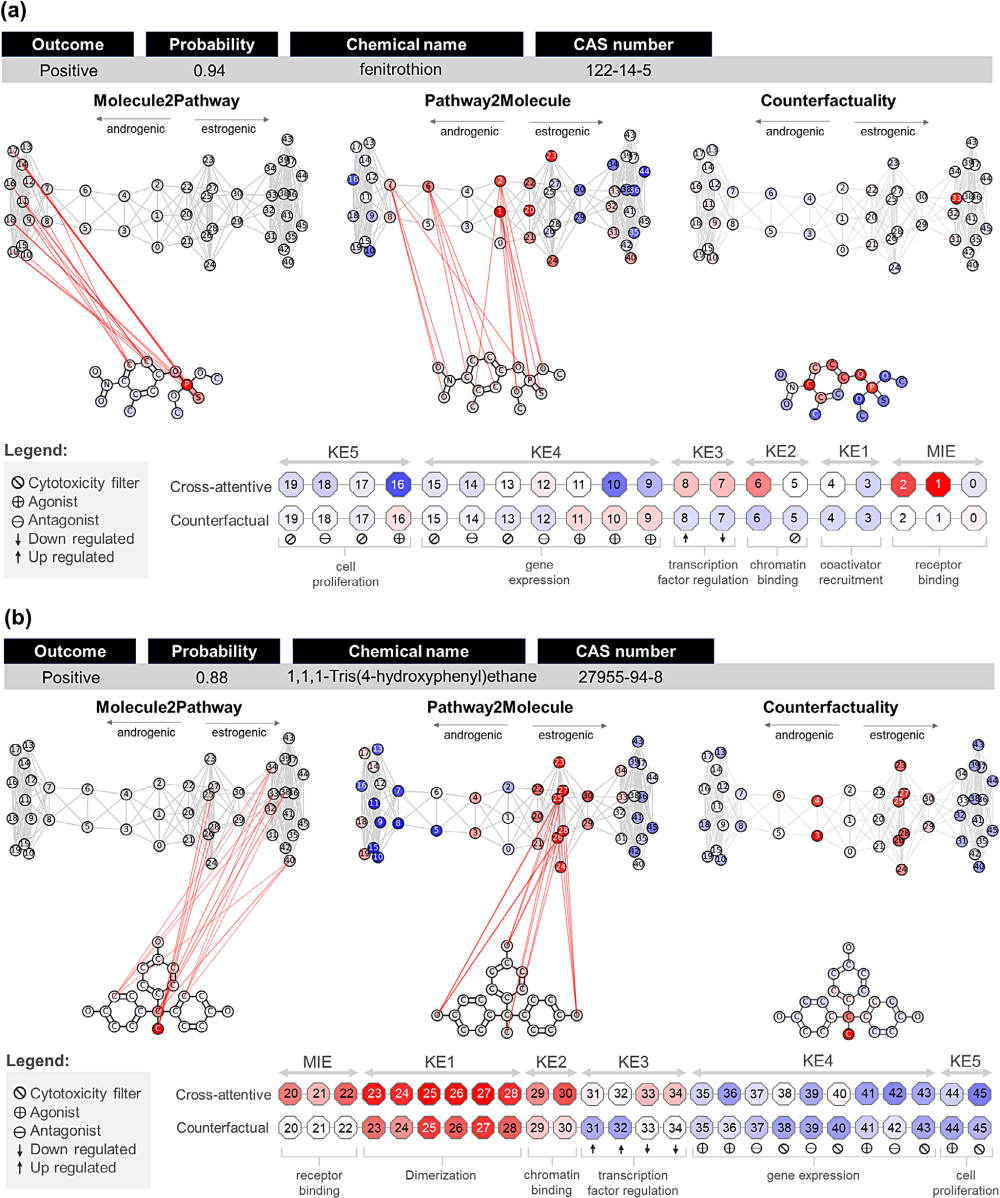
Integrative cross‐attention (non‐causal) and counterfactual analyses illustrating the mechanistic interpretability of Tier‐2 predictions for (a) fenitrothion (Hershberger positive) and (b) THPE (uterotrophic positive).

To illustrate how the Tier‐2 framework provides mechanistic interpretability for endocrine disruption predictions, we first examined two representative compounds (Figure 5): fenitrothion (CAS no. 122‐14‐5), predicted as positive (probability = 0.94) in the Hershberger assay, and 1,1,1‐tris(4‐hydroxyphenyl)ethane (THPE, CAS no. 27955‐94‐8), predicted as positive (probability = 0.88) in the uterotrophic assay.

The multimodal GAT model's explainability of fenitrothion as a positive endocrine is fully consistent with its experimentally established mechanism as a competitive AR antagonist. Fenitrothion has been shown to exhibit antagonist potency comparable to the pharmaceutical antiandrogen flutamide and to surpass known environmental antiandrogens such as linuron and p,p'‐DDE in antagonist in vitro assays [72]. As shown in Figure 5a, P2M analysis identified strong associations between the molecule's thiophosphoryl and aromatic moieties and several nodes within the AR‐mediated pathway. This analysis assigned a strong positive influence (red nodes) to early‐stage events, including the receptor binding (MIE), chromatin binding (KE2), and transcription factor regulation (KE3). This pattern reflects the compound's ability to engage the AR signaling axis from its initial steps, a behavior aligned with its known role as a competitive AR antagonist. However, it is worth noting that our prediction model does not consider the compound's metabolic activation, which may be relevant for some organophosphate pro‐toxicants. On the other hand, the M2P and counterfactuality maps show a robust concentration of influence specifically linking the molecule's thiophosphoryl group to androgen‐dependent gene expression assays (KE4) and to cell‐proliferation outcomes (KE5), both of which display strong antagonistic signatures characterized by pathway inhibition rather than activation.

The multimodal AttentiveFP model's explainability of THPE as a positive endocrine disruptor in the uterotrophic assay is the mode of action. This alignment reflects the presence of para‐hydroxylated aromatic rings, structural motifs commonly associated with ER agonism. As shown in Figure 5b, the P2M analysis identified strong associations between these aromatic hydroxyl groups and multiple nodes across the ER‐mediated pathway, highlighting them as the key structural determinants driving the model's prediction. This analysis assigned a pronounced positive influence (red nodes) to early‐stage events, particularly the ER binding (MIE), receptor dimerization (KE1), and chromatin binding (KE2). A similar pattern was observed in the counterfactuality map, which likewise emphasized these early pathway events as the principal drivers of the compound's positive prediction. These results suggest that the compound prediction was impacted by directed ER‐mediated effects, a behavior characteristic of bisphenols [73, 74, 75, 76].

In contrast, the M2P map assigned greater attention to the THPE's quaternary carbon, linking it to more advanced stages of the AOP. This complementary behavior shows that the bidirectional cross‐attention mechanism attends to distinct regions of the pathway: P2M focuses on early initiating events, and M2P captures downstream biological responses. At KE3, the model highlighted associations with transcription factor downregulation, a pattern consistent with an antagonist‐like activity. At KE4, the associations were directed toward cytotoxicity‐filter assays within the antagonist panel, further indicating that the model captured downstream perturbations. These observations are consistent with in vivo findings for THPE, which has been shown to exert antiestrogenic effects in uterine‐development assays in mice, including suppressed uterine growth and downregulation of estrogen‐responsive genes at low doses [77].

## 6 Conclusion

This work introduces an innovative multimodal cross‐attentive framework that fuses chemical graph information with AOP‐anchored, assay‐level signals to predict organism‐level endocrine outcomes. The multi‐tier design, composed of multitask Tier‐1 models that learn AR/ER MIEs and KEs, followed by Tier‐2 cross‐attentive models, achieved high and consistent predictive performance for both Hershberger and uterotrophic endpoints. It outperformed single‐source baselines in ablation studies and exhibited strong generalization in retrospective analyses. The framework also produces interpretable predictions by revealing how molecular substructures align with AR‐ and ER‐mediated events across the AOP (MIE→KE5). This bidirectional perspective clarifies how specific chemical motifs propagate into and modulate pathway‐level perturbation patterns that ultimately drive each in vivo decision. In summary, our computational framework is an IATA‐compliant tool for early virtual screening of endocrine disruptors. Developed models can substantially reduce redundant animal testing and support more efficient allocation of experimental resources. At the same time, Tier‐2 predictions should be interpreted with key limitations in mind. These include constraints imposed by the chemical space, as reflected by scaffold splitting behavior (Table S2), potential species‐ and strain‐specific differences, untested dose–response regimens, and the inability to fully represent mixture effects, cell–cell interactions, epigenetic modifications, tissue‐ and context‐dependent signaling, and higher‐order homeostatic regulation in vivo. In addition, our AR‐ and ER‐focused framework does not encompass other biologically relevant endocrine‐disruption mechanisms, such as thyroid hormone signaling, steroidogenic enzymes, retinoid pathways, and peroxisome proliferator‐activated receptor‐mediated responses. Finally, our work does not incorporate explicit toxicokinetic properties, which may substantially influence endocrine disruption in vivo. In summary, our framework offers a practical and mechanistically grounded path toward more efficient screening and prioritization within modern IATA‐based regulatory programs.

## Author Contributions

The manuscript was written through the contributions of all authors. All authors have given approval to the final version of the manuscript.

## Funding

This study received financial support from the FAPEG (grant #202310267001412, #202510267001513), CAPES (Finance Code 001, AUXPE number 88881.845026/2023‐01), and CNPq (grant #408678/2024‐0). B.J.N. is CNPq productivity fellow (grant #311100/2023‐6). The funders had no role in the design and conduct of the study, the collection, management, analysis, and interpretation of the data, the preparation, review, or approval of the manuscript, or the decision to submit the manuscript for publication.

## Conflicts of Interest

R.C.B. and E.N.M. are co‐founders of InsilicAll Ltda. and Predictive LLC, respectively, which develop novel alternative methods and software for toxicity prediction. All the other authors declare no conflicts.

## Supporting information




**Supporting File**: advs74254‐sup‐0001‐SuppMat.docx.

## Data Availability

The data and code that support the findings of this study are openly available in the following GitHub repositories: Tier‐1 at [https://github.com/LCi‐UFG/HolisticGNN] and Tier‐2 at [https://github.com/LCi‐UFG/PredictED].

## References

[1] E. K. Shanle and W. Xu , “Endocrine Disrupting Chemicals Targeting Estrogen Receptor Signaling: Identification and Mechanisms of Action,” Chemical Research in Toxicology 24, no. 1 (2011): 6–19, 10.1021/tx100231n. 21053929 PMC3119362

[2] R. Rizzo , D. Bortolotti , S. Rizzo , and G. Schiuma , (ed. R. Marci )“Cellular Mechanisms of Endocrine Disruption,” Environment Impact on Reproductive Health: A Translational Approach, (Springer International Publishing, 2023): pp. 15–48, 10.1007/978-3-031-36494-5_2.

[3] C. Ahn and E.‐B. Jeung , “Endocrine‐Disrupting Chemicals and Disease Endpoints,” International Journal of Molecular Sciences 24, no. 6 (2023): 5342, 10.3390/ijms24065342. 36982431 PMC10049097

[4] S. M. Cripps , S. A. Marshall , D. M. Mattiske , R. Y. Ingham , and A. J. Pask , “Estrogenic Endocrine Disruptor Exposure Directly Impacts Erectile Function,” Communications Biology 7, no. 1 (2024): 403, 10.1038/s42003-024-06048-1. 38565966 PMC10987563

[5] I. A. Kawa , A. Masood , Q. Fatima , et al., “Endocrine Disrupting Chemical Bisphenol A and its potential effects on Female Health,” Diabetes & Metabolic Syndrome: Clinical Research & Reviews 15, no. 3 (2021): 803–811, 10.1016/j.dsx.2021.03.031. 33839640

[6] A. Lacouture , C. Lafront , C. Peillex , M. Pelletier , and É. Audet‐Walsh , “Impacts of Endocrine‐Disrupting Chemicals on Prostate Function and Cancer,” Environmental Research 204 (2022): 112085, 10.1016/j.envres.2021.112085. 34562481

[7] A. M. Soto and C. D. D. T. Sonnenschein , “DDT, Endocrine Disruption and Breast Cancer,” Nature Reviews Endocrinology 11, no. 9 (2015): 507–508, 10.1038/nrendo.2015.125. PMC4667778 26239610

[8] L. Trasande , R. T. Zoeller , U. Hass , et al., “Estimating Burden and Disease Costs of Exposure to Endocrine‐Disrupting Chemicals in the European Union,” The Journal of Clinical Endocrinology & Metabolism 100, no. 4 (2015): 1245–1255, 10.1210/jc.2014-4324. 25742516 PMC4399291

[9] M. K. Manibusan and L. W. Touart , “A Comprehensive Review of Regulatory Test Methods for Endocrine Adverse Health Effects,” Critical Reviews in Toxicology 47, no. 6 (2017): 440–488, 10.1080/10408444.2016.1272095. 28617201

[10] A. M. Richard , R. S. Judson , K. A. Houck , et al., “ToxCast Chemical Landscape: Paving the Road to 21st Century Toxicology,” Chemical Research in Toxicology 29, no. 8 (2016): 1225–1251, 10.1021/acs.chemrestox.6b00135. 27367298

[11] A. M. Richard , R. Huang , S. Waidyanatha , et al., “The Tox21 10K Compound Library: Collaborative Chemistry Advancing Toxicology,” Chemical Research in Toxicology 34, no. 2 (2021): 189–216, 10.1021/acs.chemrestox.0c00264. 33140634 PMC7887805

[12] J. Jeong , D. Kim , and J. Choi , “Application of ToxCast/Tox21 Data for Toxicity Mechanism‐Based Evaluation and Prioritization of Environmental Chemicals: Perspective and Limitations,” Toxicology in Vitro 84 (2022): 105451, 10.1016/j.tiv.2022.105451. 35921976

[13] N. C. Kleinstreuer , P. Ceger , E. D. Watt , et al., “Development and Validation of a Computational Model for Androgen Receptor Activity,” Chemical Research in Toxicology 30, no. 4 (2017): 946–964, 10.1021/acs.chemrestox.6b00347. 27933809 PMC5396026

[14] R. S. Judson , F. M. Magpantay , V. Chickarmane , et al., “Integrated Model of Chemical Perturbations of a Biological Pathway Using 18 in Vitro High‐Throughput Screening Assays for the Estrogen Receptor,” Toxicological Sciences 148, no. 1 (2015): 137–154, 10.1093/toxsci/kfv168. 26272952 PMC4635633

[15] R. S. Thomas , R. S. Paules , A. Simeonov , et al., “The US Federal Tox21 Program: A Strategic and Operational Plan for Continued Leadership,” Altex 35, no. 2 (2018): 163–168, 10.14573/altex.1803011. 29529324 PMC6664816

[16] D. M. Rotroff , D. J. Dix , K. A. Houck , et al., “Using in Vitro High Throughput Screening Assays to Identify Potential Endocrine‐Disrupting Chemicals,” Environmental Health Perspectives 121, no. 1 (2013): 7–14, 10.1289/ehp.1205065. 23052129 PMC3546348

[17] P. Browne , P. D. Noyes , W. M. Casey , and D. J. Dix , “Application of Adverse Outcome Pathways to U.S. EPA's Endocrine Disruptor Screening Program,” Environmental Health Perspectives 125, no. 9 (2017): 096001, 10.1289/EHP1304. 28934726 PMC5915179

[18] R. S. Thomas , T. Bahadori , T. J. Buckley , et al., “The Next Generation Blueprint of Computational Toxicology at the U.S. Environmental Protection Agency,” Toxicological Sciences 169, no. 2 (2019): 317–332, 10.1093/toxsci/kfz058. 30835285 PMC6542711

[19] Y. Chen , F. Cheng , L. Sun , W. Li , G. Liu , and Y. Tang , “Computational Models to Predict Endocrine‐Disrupting Chemical Binding With Androgen or Oestrogen Receptors,” Ecotoxicology and Environmental Safety 110 (2014): 280–287, 10.1016/j.ecoenv.2014.08.026. 25282305

[20] K. Mansouri , A. Abdelaziz , A. Rybacka , et al., “CERAPP: Collaborative Estrogen Receptor Activity Prediction Project,” Environmental Health Perspectives 124, no. 7 (2016): 1023–1033, 10.1289/ehp.1510267. 26908244 PMC4937869

[21] K. Mansouri , N. Kleinstreuer , A. M. Abdelaziz , et al., “CoMPARA: Collaborative Modeling Project for Androgen Receptor Activity,” Environmental Health Perspectives 128, no. 2 (2020): 027002, 10.1289/EHP5580. 32074470 PMC7064318

[22] N. Schaduangrat , N. Anuwongcharoen , P. Charoenkwan , and W. Shoombuatong , “DeepAR: A Novel Deep Learning‐Based Hybrid Framework for the Interpretable Prediction of Androgen Receptor Antagonists,” Journal of Cheminformatics 15, no. 1 (2023): 50, 10.1186/s13321-023-00721-z. 37149650 PMC10163717

[23] H. Tan , J. Wu , R. Zhang , et al., “Development, Validation, and Application of a Human Reproductive Toxicity Prediction Model Based on Adverse Outcome Pathway,” Environmental Science & Technology 56, no. 17 (2022): 12391–12403, 10.1021/acs.est.2c02242. 35960020

[24] G. Piir , S. Sild , and U. Maran , “Interpretable Machine Learning for the Identification of Estrogen Receptor Agonists, Antagonists, and Binders,” Chemosphere 347 (2024): 140671, 10.1016/j.chemosphere.2023.140671. 37951393

[25] L. Xin , S. Liu , W. Shi , G.‐G. Ying , X. Hui , and C.‐E. Chen , “Knowledge‐based machine learning for predicting and understanding the androgen receptor (AR)‐mediated reproductive toxicity in zebrafish,” Environment International 191 (2024): 108995, 10.1016/j.envint.2024.108995. 39241331

[26] M. J. Moné , G. Pallocca , S. E. Escher , et al., “Setting the Stage for Next‐Generation Risk Assessment With Non‐Animal Approaches: The EU‐ToxRisk Project Experience,” Archives of Toxicology 94, no. 10 (2020): 3581–3592, 10.1007/s00204-020-02866-4. 32886186 PMC7502065

[27] N. Ball , R. Bars , P. A. Botham , et al., “A Framework for Chemical Safety Assessment Incorporating New Approach Methodologies Within REACH,” Archives of Toxicology 96, no. 3 (2022): 743–766, 10.1007/s00204-021-03215-9. 35103819 PMC8850243

[28] P. Reiser , M. Neubert , A. Eberhard , et al., “Graph Neural Networks for Materials Science and Chemistry,” Communications Materials 3, no. 1 (2022): 93, 10.1038/s43246-022-00315-6. 36468086 PMC9702700

[29] G. Corso , H. Stark , S. Jegelka , T. Jaakkola , and R. Barzilay , “Graph Neural Networks,” Nature Reviews Methods Primers 4, no. 1 (2024): 17, 10.1038/s43586-024-00294-7.

[30] K. Soulios , P. Scheibe , M. Bernt , J. Hackermüller , and J. D. Schor , “Deepfplearn +: Enhancing Toxicity Prediction Across the Chemical Universe Using Graph Neural Networks,” Bioinformatics 39, no. 12 (2023): btad713, 10.1093/bioinformatics/btad713. 38011648 PMC10724847

[31] L. H. M. Torres , J. P. Arrais , and B. Ribeiro , “Combining Graph Neural Networks and Transformers for Few‐Shot Nuclear Receptor Binding Activity Prediction,” Journal of Cheminformatics 16, no. 1 (2024): 109, 10.1186/s13321-024-00902-4. 39334272 PMC11429188

[32] Y. Ren , X. Wu , Z. Xi , et al., “Novel Graph Neural Network Reveals Binding Mechanisms and Environmental Risks of PAHs Interaction With Estrogen Receptor B,” Environmental Pollution 384 (2025): 127011, 10.1016/j.envpol.2025.127011. 40876732

[33] P. Browne , N. C. Kleinstreuer , P. Ceger , et al., “Development of a Curated Hershberger Database,” Reproductive Toxicology 81 (2018): 259–271, 10.1016/j.reprotox.2018.08.016. 30205136 PMC6711601

[34] N. C. Kleinstreuer , P. C. Ceger , D. G. Allen , et al., “A Curated Database of Rodent Uterotrophic Bioactivity,” Environmental Health Perspectives 124, no. 5 (2016): 556–562, 10.1289/ehp.1510183. 26431337 PMC4858395

[35] D. Fourches , E. Muratov , and A. T. Tropsha , “Trust, But Verify: On the Importance of Chemical Structure Curation in Cheminformatics and QSAR Modeling Research,” Journal of Chemical Information and Modeling 50, no. 7 (2010): 1189–1204, 10.1021/ci100176x. 20572635 PMC2989419

[36] D. Fourches , E. Muratov , and A. Tropsha , “Trust, but Verify II: A Practical Guide to Chemogenomics Data Curation,” Journal of Chemical Information and Modeling 56, no. 7 (2016): 1243–1252, 10.1021/acs.jcim.6b00129. 27280890 PMC5657146

[37] R. Joeres , D. B. Blumenthal , and O. V. Kalinina , “Data Splitting to Avoid Information Leakage With DataSAIL,” Nature Communications 16, no. 1 (2025): 3337, 10.1038/s41467-025-58606-8. PMC11978981 40199913

[38] J. Gilmer , S. S. Schoenholz , P. F. Riley , O. Vinyals , and G. E. Dahl , “Neural Message Passing for Quantum Chemistry,” arXiv (2017), 10.48550/arXiv.1704.01212.

[39] P. Veličković , G. Cucurull , A. Casanova , A. Romero , P. Liò , and Y. Bengio , “Graph Attention Networks,” arXiv (2018), 10.48550/arXiv.1710.10903.

[40] K. Xu , W. Hu , J. Leskovec , and S. Jegelka , “How Powerful Are Graph Neural Networks?,” arXiv (2019), 10.48550/arXiv.1810.00826.

[41] T. Cai , S. Luo , K. Xu , D. He , T.‐Y. Liu , and L. Wang , “GraphNorm: A Principled Approach to Accelerating Graph Neural Network Training,” arXiv (2021), 10.48550/arXiv.2009.03294.

[42] Z. Xiong , D. Wang , X. Liu , et al., “Pushing the Boundaries of Molecular Representation for Drug Discovery With the Graph Attention Mechanism,” Journal of Medicinal Chemistry 63, no. 16 (2020): 8749–8760, 10.1021/acs.jmedchem.9b00959. 31408336

[43] R. Cipolla , Y. Gal , and A. Kendall , “Multi‐Task Learning Using Uncertainty to Weigh Losses for Scene Geometry and Semantics,” in 2018 IEEE/CVF Conference on Computer Vision and Pattern Recognition , (IEEE, 2018), 7482–7491, 10.1109/CVPR.2018.00781.

[44] J. T. Moreira‐Filho , R. C. Braga , J. M. Lemos , et al., “BeeToxAI: An Artificial Intelligence‐Based Web App to Assess Acute Toxicity of Chemicals to Honey Bees,” Artificial Intelligence in the Life Sciences 1 (2021): 100013, 10.1016/j.ailsci.2021.100013.

[45] D. Rogers and M. Hahn , “Extended‐Connectivity Fingerprints,” Journal of Chemical Information and Modeling 50, no. 5 (2010): 742–754, 10.1021/ci100050t. 20426451

[46] K. Mansouri , N. Kleinstreuer , A. M. Abdelaziz , et al., “CoMPARA: Collaborative Modeling Project for Androgen Receptor Activity,” Environmental Health Perspectives 128, no. 2 (2020): 27002, 10.1289/EHP5580. 32074470 PMC7064318

[47] K. Mansouri , C. M. Grulke , R. S. Judson , and A. J. Williams , “OPERA Models for Predicting Physicochemical Properties and Environmental Fate Endpoints,” Journal of Cheminformatics 10, no. 1 (2018): 10, 10.1186/s13321-018-0263-1. 29520515 PMC5843579

[48] B. I. Escher , L. Henneberger , M. König , R. Schlichting , and F. C. Fischer , “Cytotoxicity Burst? Differentiating Specific From Nonspecific Effects in Tox21 in Vitro Reporter Gene Assays,” Environmental Health Perspectives 128, no. 7 (2020): 077007, 10.1289/EHP6664. 32700975 PMC7377237

[49] R. Judson , K. Houck , M. Martin , et al., “Editor's Highlight: Analysis of the Effects of Cell Stress and Cytotoxicity on In Vitro Assay Activity Across a Diverse Chemical and Assay Space,” Toxicological Sciences 152, no. 2 (2016): 323–339, 10.1093/toxsci/kfw092. 27208079 PMC6280881

[50] R. B. Conolly , G. T. Ankley , W. Cheng , et al., “Quantitative Adverse Outcome Pathways and Their Application to Predictive Toxicology,” Environmental Science & Technology 51, no. 8 (2017): 4661–4672, 10.1021/acs.est.6b06230. 28355063 PMC6134852

[51] E. J. Perkins , R. Ashauer , L. Burgoon , et al., “Building and Applying Quantitative Adverse Outcome Pathway Models for Chemical Hazard and Risk Assessment,” Environmental Toxicology and Chemistry 38, no. 9 (2019): 1850–1865, 10.1002/etc.4505. 31127958 PMC6771761

[52] National Toxicology Program U.S. Environmental Protection Agency. Hershberger Bioassay for Padimate‐O and Homosalate – Endocrine Disruptor Screening Program (EDSP) Tier 1 Screening (National Institute of Environmental Health Sciences (NIEHS), U.S. Department of Health and Human Services, 2012).

[53] U.S. Environmental Protection Agency The Hershberger Bioassay for Ensulizole and Avobenzone – Endocrine Disruptor Screening Program (EDSP) Tier 1 Screening (National Institute of Environmental Health Sciences (NIEHS), U.S. Department of Health and Human Services, 2012).

[54] U.S. Food and Drug Administration, Center for Drug Evaluation and Research Application Number: 204684Orig1s000 (U.S. Food and Drug Administration (FDA), 2013).

[55] A. S. Murr , A. R. Buckalew , G. Devane , et al., “Peripubertal Exposure to Oxyfluorfen, a Diphenyl Herbicide, Delays Pubertal Development in the Male Rat by Antagonizing Androgen Receptor Activity,” Toxicological Sciences 203, no. 2 (2025): 206–215, 10.1093/toxsci/kfae144. 39495161 PMC12326371

[56] U.S. Food and Drug Administration, Center for Drug Evaluation and Research Pharmacology Review for NDA 022522Orig1s000 (U.S. Food and Drug Administration, Center for Drug Evaluation and Research, 2010).

[57] U.S. Food and Drug Administration, Center for Drug Evaluation and Research Pharmacology/Toxicology Review and Evaluation for NDA 203415Orig1s000 (U.S. Food and Drug Administration, Center for Drug Evaluation and Research, 2012).

[58] U.S. Food and Drug Administration, Center for Drug Evaluation and Research Pharmacology Review for NDA 207999Orig1s000, (U.S. Food and Drug Administration, Center for Drug Evaluation and Research, 2016).

[59] Z. Shangguan , K. Yang , Q. Pan , H. Hu , and P. Wang , “Dye Intermediates N‐Phenyl‐2‐Naphthylamine and o‐Tolidine Are Novel Environmental Androgens With Reproductive Toxicity in Male Rats,” Reproductive Toxicology 136 (2025): 108955, 10.1016/j.reprotox.2025.108955. 40449632

[60] A. Casas‐Rodríguez , N. Ayala‐Soldado , A. M. Cameán , and A. Jos , “Evaluation of Potential Androgenic and Antiandrogenic Activities of Pure Cyanotoxins (Microcystin‐LR and Cylindrospermopsin) Using the Hershberger Bioassay (OECD TG 441),” Ecotoxicology and Environmental Safety 302 (2025): 118780, 10.1016/j.ecoenv.2025.118780. 40749394

[61] L. Hareng , S. N. Kolle , C. Gomes , S. Schneider , and M. Wahl , “Critical Assessment of the Endocrine Potential of Linalool and Linalyl Acetate: Proactive Testing Strategy Assessing Estrogenic and Androgenic Activity of Lavender Oil Main Components,” Archives of Toxicology 98, no. 1 (2024): 347–361, 10.1007/s00204-023-03623-z. 37906319 PMC10761525

[62] M. S. Lee , D. I. Bunin , A. M. Furimsky , et al., “Novel progestogenic androgens for male contraception: Design, synthesis, and activity of C7 α‐substituted testosterone,” Biology of Reproduction 109, no. 6 (2023): 851–863, 10.1093/biolre/ioad111. 37669128 PMC10724455

[63] C. R. Sung , H. G. Kang , J. Y. Hong , and S. J. Kwack , “Citrate Ester Substitutes for Di‐2‐Ethylhexyl Phthalate: In Vivo Reproductive and in Vitro Cytotoxicity Assessments,” Journal of Toxicology and Environmental Health, Part A 83, no. 17–18 (2020): 589–595, 10.1080/15287394.2020.1798832. 32727286

[64] T. Igarashi , S. Yokota , A. Aida , T. Nishimura , and S. Kitajima , “ *In Vivo* Screening Evaluation of 12 Chemicals as Candidate Endocrine Disruptors Using Ovariectomized Mouse Uterotrophic Bioassay,” Regulatory Toxicology and Pharmacology 162 (2025): 105900, 10.1016/j.yrtph.2025.105900. 40609822

[65] B. S. Komm , Y. P. Kharode , P. V. N. Bodine , H. A. Harris , C. P. Miller , and C. R. Lyttle , “Bazedoxifene Acetate: A Selective Estrogen Receptor Modulator With Improved Selectivity,” Endocrinology 146, no. 9 (2005): 3999–4008, 10.1210/en.2005-0030. 15961563

[66] M. T. Passoni , G. Palu , N. Grechi , et al., “Uterotrophic and *in Vitro* Screening for (Anti)Estrogenic Activity of Dipyrone,” Toxicology Letters 352 (2021): 1–8, 10.1016/j.toxlet.2021.09.004. 34536523

[67] R. Ohta , A. Takagi , H. Ohmukai , et al., “Ovariectomized Mouse Uterotrophic Assay of 36 Chemicals,” The Journal of Toxicological Sciences 37, no. 5 (2012): 879–889, 10.2131/jts.37.879. 23037998

[68] M. Riedmeier , S. Antonini , C. Benoit , et al., “Isosexual Precocious Pseudopuberty During Mitotane Treatment in a Child With Adrenocortical Carcinoma: A Case Report,” Pediatric Hematology Oncology Journal 9, no. 2 (2024): 74–77, 10.1016/j.phoj.2024.03.005.

[69] P. Carthew , R. E. Edwards , B. M. Nolan , M. J. Tucker , and L. L. Smith , “Compartmentalized Uterotrophic Effects of Tamoxifen, Toremifene, and Estradiol in the Ovariectomized Wistar (Han) Rat,” Toxicological Sciences 48, no. 2 (1999): 197–205, 10.1093/toxsci/48.2.197. 10353311

[70] Q. Qu , H. Zheng , J. Dahllund , et al., “Selective Estrogenic Effects of a Novel Triphenylethylene Compound, FC1271a, on Bone, Cholesterol Level, and Reproductive Tissues in Intact and Ovariectomized Rats1,” Endocrinology 141, no. 2 (2000): 809–820, 10.1210/endo.141.2.7342. 10650964

[71] U. K. Srivastava , “Effect of Intramuscular Norethisterone Enanthate on Compensatory Ovarian Hypertrophy in the Rat,” Indian Journal of Physiology and Pharmacology 25, no. 2 (1981): 151–157. 7287136

[72] H. Tamura , S. C. Maness , K. Reischmann , D. C. Dorman , L. E. Gray , and K. W. Gaido , “Androgen Receptor Antagonism by the Organophosphate Insecticide Fenitrothion,” Toxicological Sciences 60, no. 1 (2001): 56–62, 10.1093/toxsci/60.1.56. 11222873

[73] J. C. Gould , L. S. Leonard , S. C. Maness , et al., “Bisphenol A interacts With the estrogen receptor α in a distinct manner From estradiol,” Molecular and Cellular Endocrinology 142, no. 1–2 (1998): 203–214, 10.1016/s0303-7207(98)00084-7. 9783916

[74] A. D. Papaconstantinou , T. H. Umbreit , B. R. Fisher , P. L. Goering , N. T. Lappas , and K. M. Brown , “Bisphenol A‐Induced Increase in Uterine Weight and Alterations in Uterine Morphology in Ovariectomized B6C3F1 Mice: Role of the Estrogen Receptor,” Toxicological Sciences 56, no. 2 (2000): 332–339, 10.1093/toxsci/56.2.332. 10910991

[75] A. Matsushima , X. Liu , H. Okada , M. Shimohigashi , and Y. Shimohigashi , “Bisphenol AF Is a Full Agonist for the Estrogen Receptor ERα but a Highly Specific Antagonist for ERβ,” Environmental Health Perspectives 118, no. 9 (2010): 1267–1272, 10.1289/ehp.0901819. 20427257 PMC2944088

[76] C. Park , H. Song , J. Choi , et al., “The Mixture Effects of Bisphenol Derivatives on Estrogen Receptor and Androgen Receptor,” Environmental Pollution 260 (2020): 114036, 10.1016/j.envpol.2020.114036. 31995776

[77] H. Xiao , Y. Wang , X. Jia , et al., “Tris(4‐Hydroxyphenyl)Ethane (THPE), a Trisphenol Compound, Is Antiestrogenic and Can Retard Uterine Development in CD‐1 Mice,” Environmental Pollution 260 (2020): 113962, 10.1016/j.envpol.2020.113962. 32004960

