# Supplementary material for: Multimodal Cross‐Attentive Graph‐Based Framework for Predicting In Vivo Endocrine Disruptors
Source: Adv Sci (Weinh). 2026 Feb 15;13(21):e19897. doi: 10.1002/advs.202519897 (PMC13073300; doi:10.1002/advs.202519897)
Supplement: Supplementary file 1 — Supporting File: advs74254‐sup‐0001‐SuppMat.docx. [file ADVS-13-e19897-s001.docx]

**Supporting Information**

**Multimodal Cross-Attentive Graph-Based Framework for Predicting *In Vivo* Endocrine Disruptors**

Eder Soares de Almeida Santos,^1^ Gustavo Felizardo Santos Sandes,^1^ Artur Christian Garcia da Silva,^2^ Holli-Joi Martin,^3^ Eugene N. Muratov,^3^ Rodolpho de Campos Braga,^4^ Bruno Junior Neves^1,^*

^1^ Laboratory of Cheminformatics, Faculty of Pharmacy, Universidade Federal de Goiás, Goiás, 74605-170, Brazil

^2^ Laboratory of Education and Research in *In Vitro* Toxicology, Faculty of Pharmacy, Universidade Federal de Goiás, Goiás, 74605-170, Brazil

^3^ Laboratory for Molecular Modeling, UNC Eshelman School of Pharmacy, University of North Carolina at Chapel Hill, North Carolina, 27599-7360, USA

^4^ InsilicAll Ltda., São Paulo, 04571-010, Brazil

*Author for correspondence: [brunoneves@ufg.br](mailto:brunoneves@ufg.br)

Number of pages: 17

Number of supporting texts: 02

Number of supporting tables: 06

**Materials and Methods**

**Appendix A - Parameters of the models used in Tier-1 predictions.**

*MPNN* *model*. The Tier-1 multitask MPNN was configured with 5 message-passing layers, with hidden dimensions of 25, 116, 386, 388, and 54 units, respectively. Following the message-passing phase, a four-layer feedforward network was applied, with hidden dimensions of 250, 83, 301, and 328 units, respectively. The architecture employed the ELU activation function throughout and incorporated a dropout rate of 0.236 to mitigate overfitting. Virtual node embeddings, residual skip connections, and Jumping Knowledge mechanisms were included to enhance chemical semantic representation and alleviate oversmoothing. Training was performed using the Adam optimizer with a weight decay of 0.000365 to encourage regularization. To calibrate predictions under severe class imbalance, a probabilistic threshold of 0.117 ± 0.085 was used for binary classification.

*GIN model.* Implemented with 5 aggregation layers, presenting hidden dimensions of 95, 105, 370, 490, and 429 units, respectively. Each GIN aggregation block incorporated the learnable ε-term (ε = 0.5799), allowing adaptive control of neighborhood aggregation strength. Following the graph-level readout, a four-layer feedforward network was applied, with hidden dimensions of 177, 431, 412, and 153 units, respectively. The model used the SELU activation function throughout and adopted a dropout rate of 0.592 to reduce overfitting. Optimization was conducted using the Adam optimizer with a weight decay of 0.0004827.

*GAT model*. It consisted of 4 graph-attention layers, with hidden dimensions of 319, 166, 252, and 208 units, respectively, each employing three attention heads to refine atom-level contextualization through multi-head attention. After attention-based message passing, a three-layer feedforward network was applied, with hidden dimensions of 498, 326, and 38 units, respectively. The model used the SELU activation function and incorporated a dropout rate of 0.209 throughout the architecture. Training was performed with the Adam optimizer and a weight decay of 0.0006606 to ensure adequate regularization.

*AttentiveFP model*. The model was constructed with two attention-based message-passing layers with hidden dimensions of 398 and 323 units, respectively, followed by three attentive fingerprint timesteps to refine molecular embeddings iteratively. After graph representation learning, a four-layer feedforward network was used, with hidden dimensions of 473, 372, 405, and 10 units, respectively. The architecture employed the LeakyReLU activation function and used a dropout rate of 0.568. Model training relied on the Adam optimizer with a weight decay of 0.0001093.

**Appendix B - Parameters of the models used in Tier-2 Hershberger and Uterotrophic predictions.**

**Tier-2 Hershberger Models**

*GIN model.* The GIN model employed three aggregation layers with hidden dimensions of 96, 240, and 12 units, followed by a two-layer feedforward network with 393 and 286 units. GELU activation, a dropout rate of 0.256, six co-heads, the RAdam optimizer, and a weight decay of 2.29 × 10⁻⁷ were used.

*MPNN model*. The MPNN architecture included three message-passing layers with hidden dimensions of 72, 192, and 480 units, and a two-layer feedforward block with 432 and 323 units. ReLU activation, 0.114 dropout, three co-heads, the RAdam optimizer, and a weight decay of 7.54 × 10⁻⁷ were used.

*AttentiveFP model*. The AttentiveFP model comprised three aggregation layers (348, 48, and 300 units), two dense layers with 95 and 287 units, and two attention timesteps. GELU activation, 0.104 dropout, two co-heads, RAdam optimization, and a weight decay of 2.96 × 10⁻⁵ were employed.

*GAT model*. The Tier-2 GAT-based model for the Hershberger assay was configured with 4 message-passing (aggregation) layers, using hidden dimensions of 144, 300, 12, and 24 units, respectively. This aggregation block was followed by a four-layer feedforward network with hidden dimensions of 290, 368, 486, and 131 units, respectively. The SELU activation function was employed throughout the architecture, and a dropout rate of 0.138 was applied to reduce overfitting. The graph-attention module employed eight attention heads, complemented by two cross-attention heads to support multimodal information exchange between molecular graphs and AR-pathway graphs. Model training was performed using the RAdam optimizer with a weight decay of 9.59 × 10⁻⁶ to promote smooth regularization during optimization. A probability threshold of 0.08 ± 0.075 was applied to optimize classification performance.

**Tier-2 Uterotrophic Models**

*GIN model*. The model used three aggregation layers with hidden dimensions of 372, 372, and 36 units, followed by a four-layer feedforward module with 146, 162, 68, and 122 units. ELU activation, a dropout rate of 0.194, six co-heads, the RAdam optimizer, and a weight decay of 3.19 × 10⁻⁷ were applied.

*GAT model*. The model consisted of four aggregation layers (60, 516, 108, and 120 units) with four attention heads, and a four-layer feedforward network with 317, 235, 222, and 350 units. ReLU activation, 0.126 dropout, three co-heads, RAdam optimization, and a weight decay of 6.92×10⁻⁶ were used.

*MPNN model*. The architecture replicated the uterotrophic GIN configuration, with three aggregation layers (372, 372, 36 units) and four dense layers (146, 162, 68, 122 units). ELU activation, 0.194 dropout, six co-heads, RAdam optimization, and a weight decay of 3.19×10⁻⁷ were employed.

*Tier-2 AttentiveFP model*. The Tier-2 AttentiveFP-based model for the uterotrophic assay was configured with two message-passing (aggregation) layers, each with 120 and 60 hidden units, respectively. This aggregation module was followed by a four-layer feedforward network with hidden dimensions of 485, 22, 373, and 138 units, respectively. The architecture employed the GELU activation function throughout, together with a dropout rate of 0.133 to mitigate overfitting. The model used two attentive message-passing timesteps and incorporated three cross-attention heads to integrate information between molecular graphs and ER-pathway graphs. Training was performed with the RAdam optimizer and a weight decay of 1.22 × 10⁻⁶, resulting in stable optimization and improved generalization. A probability threshold of 0.50 was used for final classification.

**Table S1.** Summary of AR- and ER-mediated assays from ToxCast/Tox21 database.

| **Task index** | **Assay** | **AOP** | **Description** | **Organism** | **Tissue** | **Cell** |
| --- | --- | --- | --- | --- | --- | --- |
| 0 | NVS_NR_cAR | MIE | NVS_NR_cAR is a biochemical, single-readout assay that uses extracted gene-proteins from Sf9/Sf21 in a cell-free assay. Measurements were taken 72 hours after chemical dosing in a 96-well plate. It is designed to measure radioligand binding using Lysate-based radiodetection technology and scintillation counting. Changes to scintillation counting signals produced from the receptor-ligand binding of the key ligand [[3H]-methyltrienolone] are indicative of a change in receptor function and kinetics for the chimpanzee androgen receptor. | Chimpanzee | NA | NA |
| 1 | NVS_NR_rAR | MIE | NVS_NR_rAR is a biochemical, single-readout assay that uses extracted gene-proteins from Testosterone pre-treated rat prostate in a tissue-based cell-free assay. Measurements were taken 18 hours after chemical dosing in a 96-well plate. It is designed to measure radioligand binding using a binding reporter, with Filter-based radiodetection technology and scintillation counting. Changes to scintillation counting signals produced from the receptor-ligand binding of the key ligand [[3H]-methyltrienolone] are indicative of a change in receptor function and kinetics for the Norway rat androgen receptor. | Rat | Prostate | NA |
| 2 | NVS_NR_hAR | MIE | NVS_NR_hAR is a biochemical, single-readout assay that uses extracted gene-proteins from LnCAP in a cell-free assay. Measurements were taken 20 hours after chemical dosing in a 96-well plate. It is designed to measure radioligand binding using Lysate-based radiodetection technology and scintillation counting. Changes to scintillation counting signals produced from the receptor-ligand binding of the key ligand [[3H]-methyltrienolone] are indicative of a change in receptor function and kinetics for the human androgen receptor. | Human | NA | NA |
| 3 | OT_AR_ARSRC1_0480 | KE1 | OT_AR_ARSRC1_0480 is a cell-based, single-readout assay that uses HEK293T, a human kidney cell line, with measurements taken at 8 hours after chemical dosing in a 384-well plate. It is designed to measure protein fragment complementation, a form of binding reporter, using fluorescence intensity signals in Protein-fragment Complementation technology. Changes in fluorescence intensity signals from protein fragment complementation are indicative of changes in receptor function and kinetics for the human androgen receptor and the SRC proto-oncogene, non-receptor tyrosine kinase. | Human | Kidney | HEK293T |
| 4 | OT_AR_ARSRC1_0960 | KE1 | OT_AR_ARSRC1_0960 is a cell-based, single-readout assay that uses HEK293T, a human kidney cell line, with measurements taken at 16 hours after chemical dosing in a 384-well plate. It is designed to measure protein fragment complementation, a form of binding reporter, using fluorescence intensity signals in Protein-fragment Complementation technology. Changes in fluorescence intensity signals from protein fragment complementation are indicative of changes in receptor function and kinetics for the human androgen receptor and the SRC proto-oncogene, non-receptor tyrosine kinase. | Human | Kidney | HEK293T |
| 5 | TOX21_ARE_BLA_agonist_viability | KE2 | TOX21_ARE_BLA_Agonist is a cell-based, single-readout assay that uses HepG2, a human liver cell line, with measurements taken 24 hours after chemical dosing in a 1536-well plate. See tox21-are-bla-p1. TOX21_ARE_BLA_Agonist_viability is an assay readout measuring cellular ATP content and detected with CellTiter-Glo Luciferase-coupled ATP quantitation. Changes in bioluminescence signals produced by an enzymatic reaction catalyzed by luciferase between the key substrate and the target cofactor [ATP] are correlated with the system's viability. | Human | Liver | HepG2 |
| 6 | TOX21_ARE_BLA_agonist_ratio | KE2 | TOX21_ARE_BLA_Agonist is a cell-based, single-readout assay that uses HepG2, a human liver cell line, with measurements taken 24 hours after chemical dosing in a 1536-well plate. See tox21-are-bla-p1. TOX21_ARE_BLA_Agonist_ratio is an assay readout measuring reporter gene via receptor activity and designed using an inducible reporter (beta-lactamase induction) detected with GAL4 beta-lactamase reporter gene. The signal is derived from the ratio of cleaved (ch2) to uncleaved (ch1) reporter gene substrate used as the measure of target activity. TOX21_ARE_BLA_Agonist_ratio was designed to target transcription factor activity, specifically mapping to the NFE2L2 gene(s) using a positive control, Beta-Naphthoflavone. | Human | Liver | HepG2 |
| 7 | ATG_AR_TRANS_dn | KE3 | ATG_TRANS is a cell-based, multiplexed-readout assay that uses HepG2, a human liver cell line, with measurements taken at 24 hours after chemical dosing in a 24-well plate. It is designed to measure mRNA induction using an inducible reporter, detected by fluorescence intensity via Reverse transcription polymerase chain reaction (RT-PCR) and Capillary electrophoresis. Changes in fluorescence intensity signals indicate inducible changes in transcription factor activity. This is quantified by the level of the mRNA reporter sequence specific to the transfected trans-acting reporter gene and the exogenous transcription factor GAL4-AR, also known as the human androgen receptor. | Human | Liver | HepG2 |
| 8 | ATG_AR_TRANS_up |  |  |  |  |  |
| 9 | OT_AR_ARELUC_AG_1440 | KE4 | OT_AR_ARELUC_AG_1440 is a cell-based, single-readout assay that uses CHO-K1, a Chinese hamster ovary cell line, with measurements taken at 24 hours after chemical dosing in a 384-well plate. It is designed to measure luciferase induction, a form of inducible reporter, using bioluminescence signals from Luciferase technology. Changes to bioluminescence signals produced from an enzymatic reaction involving the key substrate [D-luciferin] are indicative of changes in transcriptional gene expression due to agonist activity regulated by the human androgen receptor. | Chinese hamster | Ovary | CHO-K1 |
| 10 | TOX21_AR_BLA_Agonist_ratio | KE4 | TOX21_AR_BLA_Agonist is a cell-based, single-readout assay that uses HEK293T, a human kidney cell line, with measurements taken at 24 hours after chemical dosing in a 1536-well plate. TOX21_AR_BLA_Agonist_ratio is an assay readout measuring reporter gene via receptor activity and designed using an inducible reporter (beta-lactamase induction) detected with GAL4 beta-lactamase reporter gene. The signal is derived from the ratio of cleaved (ch2) to uncleaved (ch1) substrate used as the measure of target activity. TOX21_AR_BLA_Agonist_ratio was designed to target nuclear receptor activity at the protein (receptor) level, specifically mapping to AR gene(s) using a positive control of R1881. | Human | Kidney | HEK293T |
| 11 | TOX21_AR_LUC_MDAKB2_Agonist | KE4 | TOX21_AR_LUC_MDAKB2_Agonist is a cell-based, single-readout assay that uses MDA-kb2, a human breast cell line, with measurements taken at 24 hours after chemical dosing in a 1536-well plate. It is designed to measure luciferase induction, a form of inducible reporter, using bioluminescence signals detected by CellTiter-Glo Luciferase-coupled ATP quantitation technology. Changes to bioluminescence signals produced from an enzymatic reaction involving the key substrate are indicative of changes in transcriptional gene expression due to agonist activity regulated by the human androgen receptor. | Human | Breast | MDA-kb2 |
| 12 | TOX21_AR_BLA_Antagonist_ratio | KE4 | TOX21_AR_BLA_Antagonist is a cell-based, single-readout assay that uses HEK293T, a human kidney cell line, with measurements taken at 24 hours after chemical dosing in a 1536-well plate. TOX21_AR_BLA_Antagonist_ratio is an assay readout measuring reporter gene via receptor activity and designed using an inducible reporter (beta-lactamase induction) detected with GAL4 beta-lactamase reporter gene. The signal is derived from the ratio of cleaved (ch2) to uncleaved (ch1) reporter gene substrate used as the measure of target activity. TOX21_AR_BLA_Antagonist_ratio was designed to target nuclear receptor activity at the protein (receptor) level, specifically mapping to AR gene(s) using a positive control of Cyproterone acetate. | Human | Kidney | HEK293T |
| 13 | TOX21_AR_BLA_Antagonist_viability | KE4 | TOX21_AR_BLA_Antagonist is a cell-based, single-readout assay that uses HEK293T, a human kidney cell line, with measurements taken at 24 hours after chemical dosing in a 1536-well plate. TOX21_AR_BLA_Antagonist_viability is an assay readout measuring cellular ATP content and detected with CellTiter-Glo Luciferase-coupled ATP quantitation. Changes in bioluminescence signals produced by an enzymatic reaction catalyzed by luciferase between the key substrate and the target cofactor correlate with the system's viability. | Human | Kidney | HEK293T |
| 14 | TOX21_AR_LUC_MDAKB2_Antagonist | KE4 | TOX21_AR_LUC_MDAKB2_Antagonist_0.5nM_R1881 is a cell-based, single-readout assay that uses MDA-kb2, a human breast cell line, with measurements taken at 24 hours after chemical dosing in a 1536-well plate. TOX21_AR_LUC_MDAKB2_Antagonist_0.5nM_R1881 is one of the assay component(s) measured or calculated from the TOX21_AR_LUC_MDAKB2_Antagonist_0.5nM_R1881. It is designed to measure luciferase induction, a form of inducible reporter, as detected by bioluminescence signals following the addition of the luciferin substrate and ATP. | Human | Breast | MDA-kb2 |
| 15 | TOX21_AR_LUC_MDAKB2_Antagonist_viability | KE4 | TOX21_AR_LUC_MDAKB2_Antagonist_0.5nM_R1881 is a cell-based, single-readout assay that uses MDA-kb2, a human breast cell line, with measurements taken at 24 hours after chemical dosing in a 1536-well plate. TOX21_AR_LUC_MDAKB2_Antagonist_0.5nM_R1881_viability used a type of viability reporter where loss-of-signal activity can be used to understand changes in cell viability. Furthermore, this assay endpoint can be considered a secondary readout, as the assay produces multiple endpoints, of which this serves a viability function. To generalize the intended target to other related targets, this assay endpoint is annotated to the cell cycle intended target family, with the subfamily cytotoxicity. | Human | Breast | MDA-kb2 |
| 16 | ACEA_AR_agonist_80hr | KE5 | ACEA_AR_agonist is a cell-based, single-readout assay that uses 22Rv1, a human prostate cancer cell line, with measurements taken at 80 hours after chemical dosing in a 384-well plate, although TO5 and TO6 (mc0.srcf) used a 96-well plate. Differences in plate size can be ignored given data normalization. ACEA_AR_agonist_80hr is one of two assay components measured or calculated from the ACEA_AR assay. It is designed to measure real-time cell-growth kinetics using a growth reporter, as detected via electrical impedance signals with Real-Time Cell Electrode Sensor (RT-CES) technology. Electrical impedance is used to quantify changes in cell growth, with higher impedance indicating greater growth. | Human | Prostate | 22Rv1 |
| 17 | ACEA_AR_agonist_AUC_viability | KE5 | ACEA_AR_agonist is a cell-based, single-readout assay that uses 22Rv1, a human prostate cancer cell line, with measurements taken at 80 hours after chemical dosing in a 384-well plate, although TO5 and TO6 (mc0.srcf) used a 96-well plate. Differences in plate size can be ignored given data normalization. ACEA_AR_80hr is one of two assay components measured or calculated from the ACEA_AR assay. It is designed to measure real-time cell-growth kinetics using a growth reporter, as detected via electrical impedance signals with Real-Time Cell Electrode Sensor (RT-CES) technology. Electrical impedance is used to quantify changes in cell growth, with higher impedance levels positively correlated with greater cell growth. Data from the assay component ACEA_AR_AUC_viability was analyzed in the positive analysis fitting direction relative to DMSO as the negative control and baseline of activity.  Using a growth reporter, loss-of-signal activity can be used to understand changes in viability.  Furthermore, this assay endpoint can be considered a secondary readout because the assay produces multiple endpoints, of which this serves as a viability endpoint.  To generalize the intended target to other related targets, this assay endpoint is annotated to the cell cycle intended target family, with the subfamily cytotoxicity. | Human | Prostate | 22Rv1 |
| 18 | ACEA_AR_antagonist_80hr | KE5 | ACEA_AR_antagonist is a cell-based, single-readout assay that uses 22Rv1, a human prostate cancer cell line, with measurements taken at 80 hours after chemical dosing in a 384-well plate, although TO5 and TO6 (mc0.srcf) used a 96-well plate. Differences in plate size can be ignored given data normalization. ACEA_AR_antagonist_80hr is one of two assay components measured or calculated from the ACEA_AR assay. It is designed to measure real-time cell-growth kinetics using a growth reporter, as detected via electrical impedance signals with Real-Time Cell Electrode Sensor (RT-CES) technology. Electrical impedance is used to quantify changes in cell growth, with higher impedance indicating greater growth. | Human | Prostate | 22Rv1 |
| 19 | ACEA_AR_antagonist_AUC_viability | KE5 | ACEA_AR_antagonist is a cell-based, single-readout assay that uses 22Rv1, a human prostate cancer cell line, with measurements taken at 80 hours after chemical dosing in a 384-well plate, although TO5 and TO6 (mc0.srcf) used a 96-well plate. Differences in plate size can be ignored given data normalization. ACEA_AR_80hr is one of two assay components measured or calculated from the ACEA_ER assay. It is designed to measure real-time cell-growth kinetics using a growth reporter, as detected via electrical impedance signals with Real-Time Cell Electrode Sensor (RT-CES) technology. Electrical impedance is used to quantify changes in cell growth, with higher impedance levels positively correlated with greater cell growth. Data from the assay component ACEA_AR_antagonist_AUC_viability was analyzed in the positive analysis fitting direction relative to DMSO as the negative control and baseline of activity.  Using a growth reporter, loss-of-signal activity can be used to understand changes in viability.  Furthermore, this assay endpoint can be considered a secondary readout, as the assay produces multiple endpoints, of which this serves as a viability function.  To generalize the intended target to other related targets, this assay endpoint is annotated to the cell cycle intended target family, with the subfamily cytotoxicity. | Human | Prostate | 22Rv1 |
| 20 | NVS_NR_bER | MIE | NVS_NR_bER is a biochemical, single-readout assay that uses extracted gene-proteins from Bovine uterine membranes in a tissue-based cell-free assay. Measurements were taken 18 hours after chemical dosing in a 96-well plate. NVS_NR_bER is one of the assay components measured or calculated from the NVS_NR_bER assay. It is designed to measure radioligand binding using a Lysate-based radiodetection technology and scintillation counting signals. Changes to scintillation counting signals produced from the receptor-ligand binding of the key ligand [[3H]-estradiol] are indicative of a change in receptor function and kinetics for the cattle estrogen receptor 1. | Bovine | Uterus | NA |
| 21 | NVS_NR_hER | MIE | NVS_NR_hER is a biochemical, single-readout assay that uses extracted gene-proteins from MCF7 in a cell-free assay. Measurements were taken 18 hours after chemical dosing in a 96-well plate. NVS_NR_hER is one of the assay component(s) measured or calculated from the NVS_NR_hER assay. It is designed to measure radioligand binding using a Lysate-based radiodetection technology and scintillation counting signals. Changes to scintillation counting signals produced from the receptor-ligand binding of the key ligand [[3H]-estradiol] are indicative of a change in receptor function and kinetics for the human estrogen receptor 1. | Human | NA | NA |
| 22 | NVS_NR_mERa | MIE | NVS_NR_mERa is a biochemical, single-readout assay that uses extracted gene-proteins in a cell-free assay. Measurements were taken 18 hours after chemical dosing in a 96-well plate. NVS_NR_mERa is one of the assay components measured or calculated from the NVS_NR_mERa assay. It is designed to measure radioligand binding, a form of binding reporter, using Filter-based radiodetection technology and scintillation counting. Changes to scintillation counting signals produced from the receptor-ligand binding of the key ligand [[3H]-estradiol] are indicative of a change in receptor function and kinetics for the house mouse estrogen receptor 1 (alpha). | Mouse | NA | NA |
| 23 | OT_ER_ERaERa_0480 | KE1 | OT_ER_ERaERa_0480 is a cell-based assay that uses HEK293T, a human kidney cell line, with measurements taken at 8 hours after chemical dosing in a 384-well plate.  OT_ER_ERaERa_0480 is one of the assay component(s) measured or calculated from the OT_ER_ERaERa_0480 assay. It is designed to measure protein fragment complementation, a form of binding reporter, using fluorescence intensity signals in Protein-fragment Complementation technology. Changes in fluorescence intensity signals from protein fragment complementation are indicative of changes in receptor function and kinetics for the human estrogen receptor 1. | Human | Kidney | HEK293T |
| 24 | OT_ER_ERaERa_1440 | KE1 | OT_ER_ERaERa_1440 is a cell-based assay that uses HEK293T, a human kidney cell line, with measurements taken at 24 hours after chemical dosing in a 384-well plate.  OT_ER_ERaERa_1440 is one of the assay component(s) measured or calculated from the OT_ER_ERaERa_1440 assay. It is designed to measure protein fragment complementation, a form of binding reporter, using fluorescence intensity signals in Protein-fragment Complementation technology. Changes in fluorescence intensity signals from protein fragment complementation are indicative of changes in receptor function and kinetics for the human estrogen receptor 1. | Human | Kidney | HEK293T |
| 25 | OT_ER_ERaERb_0480 | KE1 | OT_ER_ERaERb_0480 is a cell-based assay that uses HEK293T, a human kidney cell line, with measurements taken at 8 hours after chemical dosing in a 384-well plate.  OT_ER_ERaERb_0480 is one of the assay components measured or calculated from the OT_ER_ERaERb_0480 assay. It is designed to measure protein fragment complementation, a form of binding reporter, using fluorescence intensity signals in Protein-fragment Complementation technology. Changes in fluorescence intensity signals from protein fragment complementation are indicative of changes in receptor function and kinetics for the human estrogen receptors 1 and 2 (ER alpha and ER beta). | Human | Kidney | HEK293T |
| 26 | OT_ER_ERaERb_1440 | KE1 | OT_ER_ERaERb_1440 is a cell-based assay that uses HEK293T, a human kidney cell line, with measurements taken at 24 hours after chemical dosing in a 384-well plate.  OT_ER_ERaERb_1440 is one of the assay components measured or calculated from the OT_ER_ERaERb_1440 assay. It is designed to measure protein fragment complementation, a form of binding reporter, using fluorescence intensity signals in Protein-fragment Complementation technology.  Changes in fluorescence intensity signals from protein fragment complementation are indicative of changes in receptor function and kinetics for the human estrogen receptors 1 and 2 (ER alpha and ER beta). | Human | Kidney | HEK293T |
| 27 | OT_ER_ERbERb_0480 | KE1 | OT_ER_ERbERb_0480 is a cell-based assay that uses HEK293T, a human kidney cell line, with measurements taken at 8 hours after chemical dosing in a 384-well plate.  OT_ER_ERbERb_0480 is one of the assay component(s) measured or calculated from the OT_ER_ERbERb_0480 assay. It is designed to measure protein fragment complementation, a form of binding reporter, using fluorescence intensity signals in Protein-fragment Complementation technology. Changes in fluorescence intensity signals from protein fragment complementation are indicative of changes in receptor function and kinetics for the human estrogen receptor 2 (ER beta). | Human | Kidney | HEK293T |
| 28 | OT_ER_ERbERb_1440 | KE1 | OT_ER_ERbERb_1440 is a cell-based assay that uses HEK293T, a human kidney cell line, with measurements taken at 24 hours after chemical dosing in a 384-well plate.  OT_ER_ERbERb_1440 is one of the assay component(s) measured or calculated from the OT_ER_ERbERb_1440 assay. It is designed to measure protein fragment complementation, a form of binding reporter, using fluorescence intensity signals in Protein-fragment Complementation technology. Changes in fluorescence intensity signals from protein fragment complementation are indicative of changes in receptor function and kinetics for the human estrogen receptor 2 (ER beta). | Human | Kidney | HEK293T |
| 29 | OT_ERa_EREGFP_0120 | KE2 | OT_ERa_GFPERaERE_0120 is a cell-based, single-readout assay that uses HeLa, a human cervix cell line, with measurements taken at 2 hours after chemical dosing in a 384-well plate. OT_ERa_GFPERaERE_0120 is one of the assay component(s) measured or calculated from the OT_ERa_GFPERaERE_0120 assay. It is designed to measure fluorescent protein induction, a form of inducible reporter, using optical microscopy: fluorescence microscopy. Changes in optical microscopy: fluorescence microscopy signals from fluorescent protein induction are indicative of changes in receptor function and kinetics for the human estrogen receptor 1. | Human | Cervix | HeLa |
| 30 | OT_ERa_EREGFP_0480 | KE2 | OT_ERa_GFPERaERE_0480 is a cell-based, single-readout assay that uses HeLa, a human cervix cell line, with measurements taken at 8 hours after chemical dosing in a 384-well plate. OT_ERa_GFPERaERE_0480 is one of the assay components measured or calculated from the OT_ERa_GFPERaERE_0480 assay. It is designed to measure fluorescent protein induction, a form of inducible reporter, using optical microscopy: fluorescence microscopy signals from Microscopy technology. Changes in optical microscopy: fluorescence microscopy signals from fluorescent protein induction are indicative of changes in receptor function and kinetics for the human estrogen receptor 1. | Human | Cervix | HeLa |
| 31 | ATG_ERa_TRANS_up | KE3 | ATG_TRANS is a cell-based, multiplexed-readout assay that uses HepG2, a human liver cell line, with measurements taken at 24 hours after chemical dosing in a 24-well plate. ATG_ERa_TRANS is one of 30 assay components measured or calculated from the ATG_TRANS assay. It is designed to measure mRNA induction using a form of inducible reporter, detected by fluorescence intensity signals via Reverse transcription polymerase chain reaction (RT-PCR) and Capillary electrophoresis technology. Changes in fluorescence intensity signals indicate inducible changes in transcription factor activity. This is quantified by the level of the mRNA reporter sequence specific to the transfected trans-acting reporter gene and the exogenous transcription factor GAL4-ERa, also known as human estrogen receptor 1. | Human | Liver | HepG2 |
| 32 | ATG_ERE_CIS_up | KE3 | ATG_CIS is a cell-based, multiplexed-readout assay that uses HepG2, a human liver cell line, with measurements taken at 24 hours after chemical dosing in a 24-well plate. ATG_ERE_CIS is one of 52 assay components measured or calculated from the ATG_CIS assay. It is designed to measure mRNA induction using a form of inducible reporter, detected by fluorescence intensity signals via Reverse transcription polymerase chain reaction (RT-PCR) and Capillary electrophoresis technology. Changes in fluorescence intensity signals indicate inducible changes in transcription factor activity. This is quantified by the level of mRNA reporter sequence unique to the cis-acting reporter gene response element ERE, which is responsive to the endogenous human estrogen receptor 1. | Human | Liver | HepG2 |
| 33 | ATG_ERa_TRANS_dn | KE3 | ATG_ERa_TRANS_dn is a cell-based, multiplexed-readout assay that uses HepG2, a human liver cell line, with measurements taken 24 hours after chemical dosing in a 24-well plate. It is designed to measure mRNA repression, as detected by fluorescence intensity signals from Reverse transcription polymerase chain reaction (RT-PCR) and Capillary electrophoresis. Changes in fluorescence intensity signals indicate decreased transcriptional activity of the exogenous transcription factor GAL4-ERα, a chimeric construct of the human estrogen receptor alpha ligand-binding domain fused to the GAL4 DNA-binding domain. | Human | Liver | HepG2 |
| 34 | ATG_ERE_CIS_dn | KE3 | ATG_ERE_CIS_dn is a cell-based, multiplexed-readout assay that uses HepG2, a human liver cell line, with measurements taken 24 hours after chemical dosing in a 24-well plate. It is designed to measure repression of endogenous transcriptional activity via estrogen response elements (EREs), a form of cis-acting reporter, using fluorescence intensity signals detected by RT-PCR and Capillary electrophoresis. Changes in fluorescence intensity signals indicate decreased transcription factor binding and repression of estrogen receptor signaling via endogenous response elements. | Human | Liver | HepG2 |
| 35 | TOX21_ERa_BLA_Agonist_ratio | KE4 | TOX21_ERa_BLA_Agonist is a cell-based, single-readout assay that uses HEK293T, a human kidney cell line, with measurements taken at 24 hours after chemical dosing in a 1536-well plate.  TOX21_ERa_BLA_Agonist_ratio is an assay readout measuring reporter gene via receptor activity and designed using an inducible reporter (beta-lactamase induction) detected with GAL4 beta-lactamase reporter gene. The signal is derived from the ratio of cleaved (ch2) to uncleaved (ch1) substrate used as the measure of target activity. TOX21_ERa_BLA_Agonist_ratio was designed to target nuclear receptor activity at the protein (receptor) level, specifically mapping to ESR1 gene(s) using a positive control of 17b-estradiol. | Human | Kidney | HEK293T |
| 36 | TOX21_ERa_LUC_BG1_Agonist^a^ | KE4 | TOX21_ERa_LUC_VM7_Agonist is a cell-based, single-readout assay that uses VM7, a human breast tissue cell line, with measurements taken at 22 hours after chemical dosing in a 1536-well plate. It is designed to measure luciferase induction, a form of inducible reporter, using bioluminescence signals detected by CellTiter-Glo Luciferase-coupled ATP quantitation technology. TOX21_ERa_LUC_VM7_Agonist was designed to measure changes to bioluminescence signals produced from an enzymatic reaction involving the key substrate. Changes indicate alterations in transcriptional gene expression regulated by the human estrogen receptor 1. | Human | Breast | VM7 |
| 37 | TOX21_ERa_BLA_Antagonist_ratio | KE4 | TOX21_ERa_BLA_Antagonist is a cell-based, single-readout assay that uses HEK293T, a human kidney cell line, with measurements taken at 24 hours after chemical dosing in a 1536-well plate.  TOX21_ERa_BLA_Antagonist_ratio is an assay readout measuring reporter gene via receptor activity and designed using an inducible reporter (beta-lactamase induction) detected with GAL4 beta-lactamase reporter gene. The signal is derived from the ratio of cleaved (ch2) to uncleaved (ch1) reporter gene substrate used as the measure of target activity. TOX21_ERa_BLA_Antagonist_ratio was designed to target nuclear receptor activity at the protein (receptor) level, specifically mapping to ESR1 gene(s) using a positive control of 4-hydroxytamoxifen. | Human | Kidney | HEK293T |
| 38 | TOX21_ERa_BLA_Antagonist_viability | KE4 | TOX21_ERa_BLA_Antagonist is a cell-based, single-readout assay that uses HEK293T, a human kidney cell line, with measurements taken at 24 hours after chemical dosing in a 1536-well plate.  TOX21_ERa_BLA_Antagonist_viability is an assay readout measuring cellular ATP content and detected with CellTiter-Glo Luciferase-coupled ATP quantitation. Changes in bioluminescence signals produced by an enzymatic reaction catalyzed by luciferase between the key substrate and the target cofactor correlate with the system's viability. TOX21_ERa_BLA_Antagonist_viability used a type of viability reporter where loss-of-signal activity can be used to understand changes in cell viability. Furthermore, this assay endpoint can be considered a secondary readout because the assay produces multiple endpoints, of which this serves as a viability endpoint. To generalize the intended target to other related targets, this assay endpoint is annotated to the cell cycle intended target family, with the subfamily cytotoxicity. | Human | Kidney | HEK293T |
| 39 | TOX21_ERa_LUC_BG1_Antagonist^a^ | KE4 | TOX21_ERa_LUC_VM7_Antagonist_0.5nM_E2 is a cell-based, single-readout assay that uses VM7, a human breast tissue cell line, with measurements taken at 22 hours after chemical dosing in a 1536-well plate. This is a secondary assay for specificity to TOX21_ERa_LUC_VM7_Antagonist_0.1nM_E2. TOX21_ERa_LUC_VM7_Antagonist_0.5nM_E2 is one of the assay components measured or calculated from the TOX21_ERa_LUC_VM7_Antagonist_0.5nM_E2 assay. It is designed to measure luciferase induction, a form of inducible reporter, using CellTiter-Glo Luciferase-coupled ATP quantitation technology and bioluminescence detection. Changes to bioluminescence signals produced from an enzymatic reaction involving the key substrate are indicative of changes in transcriptional gene expression due to antagonist activity regulated by the human estrogen receptor 1, using a positive control of 4-hydroxytamoxifen | Human | Breast | VM7 |
| 40 | TOX21_ERa_LUC_BG1_Antagonist_viability^a^ | KE4 | TOX21_ERa_LUC_VM7_Antagonist_0.5nM_E2 is a cell-based, single-readout assay that uses VM7, a human breast tissue cell line, with measurements taken at 22 hours after chemical dosing in a 1536-well plate. This is a secondary assay for specificity to TOX21_ERa_LUC_VM7_Antagonist_0.1nM_E2. TOX21_ERa_LUC_VM7_Antagonist_0.5nM_E2_viability is an assay readout measuring cellular ATP content and detected with CellTiter-Glo Luciferase-coupled ATP quantitation. Changes in bioluminescence signals produced by an enzymatic reaction catalyzed by luciferase between the key substrate and the target cofactor correlate with the system's viability. | Human | Breast | VM7 |
| 41 | TOX21_ERb_BLA_Agonist_ratio | KE4 | TOX21_ERb_BLA_Agonist is a cell-based, single-readout assay that uses HEK293T, a human kidney cell line, with measurements taken at 24 hours after chemical dosing in a 1536-well plate. TOX21_ERb_BLA_Agonist_ratio is an assay readout measuring reporter gene via receptor activity and designed using an inducible reporter (beta-lactamase induction) detected with GAL4 beta-lactamase reporter gene. The signal is derived from the ratio of cleaved (ch2) to uncleaved (ch1) reporter gene substrate used as the measure of target activity. TOX21_ERb_BLA_Agonist_ch2 was designed to target nuclear receptor activity at the protein (receptor) level, specifically mapping to the ESR2 gene(s) using a positive control of 17b-estradiol | Human | Kidney | HEK293T |
| 42 | TOX21_ERb_BLA_Antagonist_ratio | KE4 | TOX21_ERb_BLA_Antagonist is a cell-based, single-readout assay that uses HEK293T, a human kidney cell line, with measurements taken at 24 hours after chemical dosing in a 1536-well plate. TOX21_ERb_BLA_Antagonist_ratio is an assay readout measuring reporter gene via receptor activity and designed using an inducible reporter (beta-lactamase induction) detected with GAL4 beta-lactamase reporter gene. The signal is derived from the ratio of cleaved (ch2) to uncleaved (ch1) reporter gene substrate used as the measure of target activity. TOX21_ERb_BLA_Antagonist_ratio was designed to target nuclear receptor activity at the protein (receptor) level, specifically mapping to ESR2 gene(s) using a positive control of 4-hydroxytamoxifen. | Human | Kidney | HEK293T |
| 43 | TOX21_ERb_BLA_Antagonist_viability | KE4 | TOX21_ERb_BLA_Antagonist is a cell-based, single-readout assay that uses HEK293T, a human kidney cell line, with measurements taken at 24 hours after chemical dosing in a 1536-well plate. TOX21_ERb_BLA_Antagonist_viability is an assay readout measuring cellular ATP content and detected with CellTiter-Glo Luciferase-coupled ATP quantitation. Changes in bioluminescence signals produced by an enzymatic reaction catalyzed by luciferase between the key substrate and the target cofactor correlate with the system's viability. | Human | Kidney | HEK293T |
| 44 | ACEA_ER_80hr | KE5 | ACEA_ER is a cell-based, single-readout assay that uses T47D, a human breast cell line, with measurements taken at 80 hours after chemical dosing in a 96-well plate, although TO2 (mc0.srcf) used a 384-well plate. Differences in plate size can be ignored given data normalization. ACEA_ER_80hr is one of two assay components measured or calculated from the ACEA_ER assay. It is designed to measure real-time cell-growth kinetics using a growth reporter, as detected via electrical impedance signals with Real-Time Cell Electrode Sensor (RT-CES) technology. Electrical impedance is used to quantify changes in cell growth, with higher impedance indicating greater growth. | Human | Breast | T47D |
| 45 | ACEA_ER_AUC_viability | KE5 | ACEA_ER is a cell-based, single-readout assay that uses T47D, a human breast cell line, with measurements taken at 80 hours after chemical dosing in a 96-well plate, although TO2 (mc0.srcf) used a 384-well plate. Differences in plate size can be ignored given data normalization. ACEA_ER_AUC_viability is one of two assay components measured or calculated from the ACEA_ER assay. It is designed to measure real-time cell-growth kinetics using a growth reporter, as detected via electrical impedance signals with Real-Time Cell Electrode Sensor (RT-CES) technology. Electrical impedance is used to quantify changes in the growth of the cells, where increased impedance is positively correlated with increased cell growth. | Human | Breast | T47D |

^a^ Before June 2016, the cell line was referred to as BG1Luc. Later studies revealed that the line used in the assay was not the original BG-1 ovarian carcinoma cells, but rather a variant of the human breast cancer cell line MCF7. Consequently, the cell line previously named BG1Luc4E2 was redesignated as VM7Luc4E2 (“V” = variant; “M7” = MCF7).^2–4^

**Table S2.** Split compositions for random and scaffold schemes.

| **Task index** | **Assay** | **Random split** | | | | | | **Scaffold split** | | | | | |
| --- | --- | --- | --- | --- | --- | --- | --- | --- | --- | --- | --- | --- | --- |
|  |  | **Trainining** | | **Validation** | | **Test** | | **Training** | | **Validation** | | **Test** | |
|  |  | **Positive** | **Inactive** | **Positive** | **Inactive** | **Positive** | **Inactive** | **Positive** | **Inactive** | **Positive** | **Inactive** | **Positive** | **Inactive** |
| 0 | NVS_NR_cAR | 179 | 1974 | 21 | 247 | 13 | 248 | 198 | 1810 | **1** | 85 | 17 | 590 |
| 1 | NVS_NR_rAR | 111 | 2638 | 15 | 316 | 16 | 321 | 115 | 2384 | **0** | 146 | 28 | 769 |
| 2 | NVS_NR_hAR | 116 | 2054 | 18 | 248 | 13 | 251 | 136 | 1889 | **0** | 85 | 13 | 595 |
| 3 | OT_AR_ARSRC1_0480 | 213 | 1156 | 24 | 149 | 28 | 145 | 241 | 1025 | **2** | 11 | 28 | 420 |
| 4 | OT_AR_ARSRC1_0960 | 304 | 1066 | 29 | 144 | 37 | 136 | 335 | 932 | **1** | 12 | 40 | 408 |
| 5 | TOX21_ARE_BLA_agonist_viability | 498 | 4822 | 74 | 583 | 64 | 599 | 571 | 4566 | 40 | 785 | 42 | 705 |
| 6 | TOX21_ARE_BLA_agonist_ratio | 1266 | 4045 | 169 | 488 | 148 | 516 | 1349 | 3788 | 127 | 698 | 135 | 612 |
| 7 | ATG_AR_TRANS_dn | 16 | 2685 | **1** | 321 | **1** | 332 | 14 | 2425 | **1** | 155 | **3** | 783 |
| 8 | ATG_AR_TRANS_up | 31 | 2670 | **3** | 319 | **2** | 331 | 29 | 2410 | **1** | 155 | **6** | 780 |
| 9 | OT_AR_ARELUC_AG_1440 | 112 | 1256 | 17 | 156 | 14 | 159 | 99 | 1166 | **3** | 10 | 41 | 407 |
| 10 | TOX21_AR_BLA_Agonist_ratio | 373 | 5450 | 59 | 668 | 48 | 676 | 424 | 5288 | 35 | 863 | 31 | 715 |
| 11 | TOX21_AR_LUC_MDAKB2_Agonist | 280 | 5541 | 44 | 683 | 36 | 688 | 334 | 5378 | 19 | 879 | 14 | 732 |
| 12 | TOX21_AR_BLA_Antagonist_ratio | 1189 | 4630 | 162 | 564 | 148 | 575 | 1391 | 4321 | 69 | 829 | 68 | 678 |
| 13 | TOX21_AR_BLA_Antagonist_viability | 772 | 5049 | 96 | 631 | 99 | 624 | 880 | 4832 | 56 | 842 | 56 | 690 |
| 14 | TOX21_AR_LUC_MDAKB2_Antagonist | 1164 | 4392 | 144 | 540 | 145 | 550 | 1366 | 3987 | 59 | 803 | 58 | 739 |
| 15 | TOX21_AR_LUC_MDAKB2_Antagonist_viability | 536 | 5023 | 68 | 616 | 63 | 633 | 622 | 4731 | 33 | 829 | 32 | 765 |
| 16 | ACEA_AR_agonist_80hr | 115 | 1231 | 23 | 148 | 12 | 160 | 119 | 1134 | **0** | 13 | 32 | 403 |
| 17 | ACEA_AR_agonist_AUC_viability | 431 | 915 | 50 | 121 | 59 | 113 | 472 | 781 | **1** | 12 | 75 | 361 |
| 18 | ACEA_AR_antagonist_80hr | 526 | 823 | 62 | 109 | 76 | 96 | 590 | 665 | **1** | 12 | 81 | 356 |
| 19 | ACEA_AR_antagonist_AUC_viability | 506 | 843 | 60 | 111 | 69 | 103 | 561 | 694 | **2** | 11 | 80 | 357 |
| 20 | NVS_NR_bER | 99 | 1521 | 14 | 184 | 9 | 198 | 104 | 1428 | **2** | 31 | 16 | 458 |
| 21 | NVS_NR_hER | 183 | 1957 | 19 | 241 | 24 | 243 | 201 | 1748 | **4** | 83 | 24 | 631 |
| 22 | NVS_NR_mERa | 147 | 1468 | 15 | 183 | 21 | 185 | 150 | 1377 | **1** | 32 | 34 | 441 |
| 23 | OT_ER_ERaERa_0480 | 108 | 1260 | 13 | 160 | 17 | 156 | 134 | 1131 | **1** | 12 | 4 | 444 |
| 24 | OT_ER_ERaERa_1440 | 128 | 1240 | 12 | 161 | 17 | 156 | 144 | 1121 | **1** | 12 | 13 | 435 |
| 25 | OT_ER_ERaERb_0480 | 190 | 1178 | 22 | 151 | 24 | 149 | 224 | 1041 | **0** | 13 | 13 | 435 |
| 26 | OT_ER_ERaERb_1440 | 256 | 1112 | 26 | 147 | 27 | 146 | 284 | 981 | **0** | 13 | 29 | 419 |
| 27 | OT_ER_ERbERb_0480 | 192 | 1176 | 19 | 154 | 17 | 156 | 216 | 1049 | **0** | 13 | 13 | 435 |
| 28 | OT_ER_ERbERb_1440 | 197 | 1171 | 22 | 151 | 21 | 152 | 214 | 1051 | **0** | 13 | 27 | 421 |
| 29 | OT_ERa_EREGFP_0120 | 135 | 1233 | 17 | 156 | 21 | 152 | 146 | 1119 | **1** | 12 | 30 | 418 |
| 30 | OT_ERa_EREGFP_0480 | 135 | 1233 | 22 | 151 | 20 | 153 | 155 | 1110 | **0** | 13 | 25 | 423 |
| 31 | ATG_ERa_TRANS_up | 589 | 2108 | 80 | 242 | 61 | 272 | 647 | 1792 | **9** | 147 | 83 | 704 |
| 32 | ATG_ERE_CIS_up | 648 | 2048 | 87 | 235 | 75 | 258 | 688 | 1751 | 20 | 136 | 112 | 675 |
| 33 | ATG_ERa_TRANS_dn | 14 | 2687 | **3** | 319 | **4** | 329 | 13 | 2425 | **0** | 156 | 8 | 779 |
| 34 | ATG_ERE_CIS_dn | 147 | 2551 | 15 | 307 | 23 | 310 | 138 | 2300 | **8** | 148 | 43 | 744 |
| 35 | TOX21_ERa_BLA_Agonist_ratio | 325 | 5497 | 46 | 680 | 30 | 694 | 368 | 5344 | 25 | 872 | 21 | 726 |
| 36 | TOX21_ERa_LUC_BG1_Agonist^a^ | 898 | 4915 | 119 | 608 | 109 | 613 | 983 | 4729 | 88 | 809 | 78 | 669 |
| 37 | TOX21_ERa_BLA_Antagonist_ratio | 851 | 4968 | 106 | 621 | 115 | 608 | 977 | 4735 | 50 | 847 | 67 | 680 |
| 38 | TOX21_ERa_BLA_Antagonist_viability | 228 | 5596 | 35 | 692 | 26 | 697 | 264 | 5448 | 21 | 876 | 16 | 731 |
| 39 | TOX21_ERa_LUC_BG1_Antagonist^a^ | 766 | 4790 | 99 | 585 | 102 | 594 | 910 | 4441 | 40 | 822 | 38 | 759 |
| 40 | TOX21_ERa_LUC_BG1_Antagonist_viability^a^ | 461 | 5094 | 53 | 629 | 50 | 645 | 538 | 4813 | 23 | 839 | 22 | 775 |
| 41 | TOX21_ERb_BLA_Agonist_ratio | 114 | 5443 | 14 | 670 | 11 | 686 | 127 | 5223 | **6** | 856 | **8** | 789 |
| 42 | TOX21_ERb_BLA_Antagonist_ratio | 1178 | 4379 | 150 | 534 | 140 | 557 | 1333 | 4018 | 66 | 796 | 95 | 702 |
| 43 | TOX21_ERb_BLA_Antagonist_viability | 887 | 4668 | 110 | 573 | 111 | 585 | 995 | 4356 | 58 | 804 | 81 | 716 |
| 44 | ACEA_ER_80hr | 310 | 1950 | 37 | 241 | 47 | 231 | 303 | 1701 | **0** | 36 | 95 | 701 |
| 45 | ACEA_ER_AUC_viability | 795 | 1463 | 87 | 191 | 94 | 183 | 722 | 1281 | **0** | 36 | 268 | 529 |
| - | Hershberger | 54 | 624 | **7** | 79 | **8** | 78 | 48 | 541 | **0** | 179 | 21 | 65 |
| - | Uterotrophic | 628 | 50 | **4** | 81 | **9** | 76 | 64 | 559 | **0** | 154 | **0** | 87 |

**Table S3.** Aom and bond features used for molecular graph construction.

| **Feature** | **Size (dimensions)** | **Description/Position** |
| --- | --- | --- |
| **Atom features** | | |
| Atom type | 27 | H, B, C, N, O, F, Na, Mg, Al, Si, P, S, Cl, K, Ca, Ti, V, Cr, Mn, Fe, Co, Ni, Cu, Zn, As, Se, Br, and I |
| Degree | 8 | 0, 1, 2, 3, 4, 5, 6, 7 |
| Formal charge | 6 | −1, 0, +1, +2, +3, +4 |
| Hybridization | 5 | sp, sp^2^, sp^3^d^2^, sp^3^, sp^3^d |
| Aromaticity | 1 | Aromatic, non-aromatic |
| **Bond features** | | |
| Bond type | 4 | Single, double, triple, and aromatic |
| Conjugation | 1 | Binary indicator for conjugated bonds |
| Ring membership | 1 | Binary indicator for ring bonds |
| Bond stereo/atom chirality | 6 | None, any, *Z*, *E*, *R*, and *S* |

**Table S4.** Summarized statistical characteristics of multitask GNNs for predicting AR- and ER-mediated assay outcomes**.**

| **Set** | **Threshold ± SD** | **ACC ± SD** | **FNR ± SD** | **FPR ± SD** | **Recall ± SD** | **SP ± SD** | **PPV ± SD** | **NPV ± SD** | **G-mean ± SD** | **AUC ± SD** |
| --- | --- | --- | --- | --- | --- | --- | --- | --- | --- | --- |
| **MPNN (default)** | | | | | | | | | | |
| **Training** | 0.50 ± 0.000 | 0.89 ± 0.005 | 0.79 ± 0.086 | 0.02 ± 0.008 | 0.21 ± 0.086 | 0.98 ± 0.008 | 0.67 ± 0.056 | 0.90 ± 0.010 | 0.46 ± 0.086 | 0.85 ± 0.015 |
| **Validation** | 0.50 ± 0.000 | 0.88 ± 0.006 | 0.80 ± 0.058 | 0.02 ± 0.008 | 0.20 ± 0.058 | 0.98 ± 0.008 | 0.65 ± 0.078 | 0.89 ± 0.006 | 0.44 ± 0.062 | 0.84 ± 0.008 |
| **Test** | 0.50 ± 0.000 | 0.88 ± 0.007 | 0.81 ± 0.086 | 0.02 ± 0.009 | 0.19 ± 0.086 | 0.98 ± 0.009 | 0.64 ± 0.048 | 0.90 ± 0.012 | 0.44 ± 0.090 | 0.84 ± 0.015 |
| **MPNN (weighted)** | | | | | | | | | | |
| **Training** | 0.50 ± 0.000 | 0.92 ± 0.002 | 0.52 ± 0.039 | 0.02 ± 0.003 | 0.48 ± 0.039 | 0.98 ± 0.003 | 0.77 ± 0.012 | 0.93 ± 0.005 | 0.69 ± 0.028 | 0.92 ± 0.004 |
| **Validation** | 0.50 ± 0.000 | 0.89 ± 0.003 | 0.65 ± 0.045 | 0.03 ± 0.005 | 0.35 ± 0.045 | 0.97 ± 0.005 | 0.63 ± 0.020 | 0.91 ± 0.007 | 0.58 ± 0.038 | 0.86 ± 0.008 |
| **Test** | 0.50 ± 0.000 | 0.89 ± 0.005 | 0.65 ± 0.040 | 0.03 ± 0.006 | 0.35 ± 0.040 | 0.97 ± 0.006 | 0.63 ± 0.023 | 0.91 ± 0.005 | 0.58 ± 0.032 | 0.86 ± 0.007 |
| **MPNN (calibrated)** | | | | | | | | | | |
| **Training** | 0.14 ± 0.007 | 0.85 ± 0.006 | 0.19 ± 0.005 | 0.15 ± 0.007 | 0.81 ± 0.005 | 0.85 ± 0.007 | 0.44 ± 0.013 | 0.97 ± 0.001 | 0.83 ± 0.004 | 0.92 ± 0.004 |
| **Validation** | 0.12 ± 0.009 | 0.80 ± 0.011 | 0.26 ± 0.011 | 0.19 ± 0.012 | 0.74 ± 0.011 | 0.81 ± 0.012 | 0.37 ± 0.012 | 0.95 ± 0.002 | 0.77 ± 0.007 | 0.86 ± 0.008 |
| **Test** | 0.12 ± 0.009 | 0.81 ± 0.009 | 0.28 ± 0.017 | 0.18 ± 0.011 | 0.72 ± 0.017 | 0.82 ± 0.011 | 0.36 ± 0.010 | 0.95 ± 0.004 | 0.77 ± 0.006 | 0.86 ± 0.007 |
| **GAT (default)** | | | | | | | | | | |
| **Training** | 0.50 ± 0.000 | 0.88 ± 0.002 | 0.84 ± 0.048 | 0.01 ± 0.005 | 0.16 ± 0.048 | 0.99 ± 0.005 | 0.67 ± 0.035 | 0.89 ± 0.006 | 0.39 ± 0.057 | 0.84 ± 0.005 |
| **Validation** | 0.50 ± 0.000 | 0.88 ± 0.003 | 0.86 ± 0.050 | 0.01 ± 0.005 | 0.14 ± 0.050 | 0.99 ± 0.005 | 0.66 ± 0.034 | 0.89 ± 0.004 | 0.37 ± 0.062 | 0.83 ± 0.007 |
| **Test** | 0.50 ± 0.000 | 0.88 ± 0.007 | 0.86 ± 0.050 | 0.01 ± 0.005 | 0.14 ± 0.050 | 0.99 ± 0.005 | 0.65 ± 0.025 | 0.89 ± 0.006 | 0.38 ± 0.063 | 0.83 ± 0.007 |
| **GAT (weighted)** | | | | | | | | | | |
| **Training** | 0.50 ± 0.000 | 0.89 ± 0.002 | 0.75 ± 0.045 | 0.02 ± 0.005 | 0.25 ± 0.045 | 0.98 ± 0.005 | 0.71 ± 0.033 | 0.90 ± 0.006 | 0.50 ± 0.043 | 0.87 ± 0.002 |
| **Validation** | 0.50 ± 0.000 | 0.88 ± 0.000 | 0.79 ± 0.062 | 0.02 ± 0.005 | 0.21 ± 0.062 | 0.98 ± 0.005 | 0.68 ± 0.005 | 0.89 ± 0.004 | 0.45 ± 0.066 | 0.85 ± 0.009 |
| **Test** | 0.50 ± 0.000 | 0.89 ± 0.002 | 0.78 ± 0.068 | 0.02 ± 0.007 | 0.22 ± 0.068 | 0.98 ± 0.007 | 0.69 ± 0.020 | 0.90 ± 0.002 | 0.47 ± 0.069 | 0.85 ± 0.002 |
| **GAT (calibrated)** | | | | | | | | | | |
| **Training** | 0.13 ± 0.004 | 0.79 ± 0.002 | 0.23 ± 0.002 | 0.20 ± 0.002 | 0.77 ± 0.002 | 0.80 ± 0.002 | 0.35 ± 0.000 | 0.96 ± 0.001 | 0.78 ± 0.002 | 0.87 ± 0.002 |
| **Validation** | 0.14 ± 0.019 | 0.79 ± 0.015 | 0.26 ± 0.006 | 0.20 ± 0.019 | 0.74 ± 0.006 | 0.80 ± 0.019 | 0.36 ± 0.028 | 0.95 ± 0.002 | 0.77 ± 0.006 | 0.85 ± 0.009 |
| **Test** | 0.14 ± 0.019 | 0.79 ± 0.007 | 0.28 ± 0.001 | 0.20 ± 0.009 | 0.72 ± 0.001 | 0.80 ± 0.009 | 0.35 ± 0.023 | 0.95 ± 0.002 | 0.76 ± 0.004 | 0.85 ± 0.002 |
| **GIN (default)** | | | | | | | | | | |
| **Training** | 0.50 ± 0.000 | 0.88 ± 0.001 | 0.83 ± 0.016 | 0.02 ± 0.003 | 0.17 ± 0.016 | 0.98 ± 0.003 | 0.61 ± 0.024 | 0.89 ± 0.002 | 0.41 ± 0.018 | 0.83 ± 0.006 |
| **Validation** | 0.50 ± 0.000 | 0.88 ± 0.005 | 0.85 ± 0.033 | 0.02 ± 0.004 | 0.15 ± 0.033 | 0.98 ± 0.004 | 0.58 ± 0.044 | 0.89 ± 0.006 | 0.38 ± 0.044 | 0.82 ± 0.011 |
| **Test** | 0.50 ± 0.000 | 0.88 ± 0.006 | 0.86 ± 0.035 | 0.02 ± 0.005 | 0.14 ± 0.035 | 0.98 ± 0.005 | 0.58 ± 0.053 | 0.89 ± 0.003 | 0.38 ± 0.046 | 0.81 ± 0.008 |
| **GIN (weighted)** | | | | | | | | | | |
| **Training** | 0.50 ± 0.000 | 0.89 ± 0.003 | 0.73 ± 0.056 | 0.02 ± 0.006 | 0.27 ± 0.056 | 0.98 ± 0.006 | 0.67 ± 0.032 | 0.90 ± 0.006 | 0.52 ± 0.052 | 0.87 ± 0.008 |
| **Validation** | 0.50 ± 0.000 | 0.88 ± 0.006 | 0.78 ± 0.060 | 0.02 ± 0.007 | 0.22 ± 0.060 | 0.98 ± 0.007 | 0.60 ± 0.039 | 0.90 ± 0.010 | 0.46 ± 0.059 | 0.83 ± 0.009 |
| **Test** | 0.50 ± 0.000 | 0.88 ± 0.007 | 0.78 ± 0.038 | 0.02 ± 0.009 | 0.22 ± 0.038 | 0.98 ± 0.009 | 0.59 ± 0.057 | 0.90 ± 0.006 | 0.46 ± 0.037 | 0.83 ± 0.011 |
| **GIN (calibrated)** | | | | | | | | | | |
| **Training** | 0.14 ± 0.027 | 0.79 ± 0.011 | 0.22 ± 0.010 | 0.20 ± 0.013 | 0.78 ± 0.010 | 0.80 ± 0.013 | 0.36 ± 0.015 | 0.96 ± 0.002 | 0.79 ± 0.008 | 0.87 ± 0.008 |
| **Validation** | 0.14 ± 0.030 | 0.77 ± 0.018 | 0.27 ± 0.019 | 0.23 ± 0.022 | 0.73 ± 0.019 | 0.77 ± 0.022 | 0.32 ± 0.016 | 0.95 ± 0.004 | 0.75 ± 0.008 | 0.83 ± 0.009 |
| **Test** | 0.14 ± 0.030 | 0.77 ± 0.011 | 0.29 ± 0.037 | 0.22 ± 0.017 | 0.71 ± 0.037 | 0.78 ± 0.017 | 0.32 ± 0.014 | 0.95 ± 0.006 | 0.74 ± 0.012 | 0.83 ± 0.011 |
| **AttentiveFP (default)** | | | | | | | | | | |
| **Training** | 0.50 ± 0.000 | 0.87 ± 0.001 | 1.00 ± 0.000 | 0.00 ± 0.000 | 0.00 ± 0.000 | 1.00 ± 0.000 | 0.00 ± 0.000 | 0.87 ± 0.001 | 0.00 ± 0.000 | 0.78 ± 0.044 |
| **Validation** | 0.50 ± 0.000 | 0.87 ± 0.004 | 1.00 ± 0.000 | 0.00 ± 0.000 | 0.00 ± 0.000 | 1.00 ± 0.000 | 0.00 ± 0.000 | 0.87 ± 0.004 | 0.00 ± 0.000 | 0.78 ± 0.046 |
| **Test** | 0.50 ± 0.000 | 0.87 ± 0.007 | 1.00 ± 0.000 | 0.00 ± 0.000 | 0.00 ± 0.000 | 1.00 ± 0.000 | 0.00 ± 0.000 | 0.87 ± 0.007 | 0.00 ± 0.000 | 0.78 ± 0.042 |
| **AttentiveFP (weighted)** | | | | | | | | | | |
| **Training** | 0.50 ± 0.000 | 0.88 ± 0.001 | 0.93 ± 0.011 | 0.00 ± 0.001 | 0.07 ± 0.011 | 1.00 ± 0.001 | 0.66 ± 0.016 | 0.88 ± 0.001 | 0.26 ± 0.022 | 0.83 ± 0.002 |
| **Validation** | 0.50 ± 0.000 | 0.88 ± 0.004 | 0.93 ± 0.006 | 0.01 ± 0.002 | 0.07 ± 0.006 | 0.99 ± 0.002 | 0.65 ± 0.072 | 0.88 ± 0.004 | 0.25 ± 0.013 | 0.82 ± 0.008 |
| **Test** | 0.50 ± 0.000 | 0.88 ± 0.007 | 0.94 ± 0.015 | 0.01 ± 0.001 | 0.06 ± 0.015 | 0.99 ± 0.001 | 0.63 ± 0.043 | 0.88 ± 0.007 | 0.25 ± 0.028 | 0.83 ± 0.008 |
| **AttentiveFP (calibrated)** | | | | | | | | | | |
| **Training** | 0.17 ± 0.007 | 0.77 ± 0.004 | 0.25 ± 0.008 | 0.23 ± 0.006 | 0.75 ± 0.008 | 0.77 ± 0.006 | 0.32 ± 0.003 | 0.95 ± 0.001 | 0.76 ± 0.002 | 0.83 ± 0.002 |
| **Validation** | 0.18 ± 0.010 | 0.77 ± 0.016 | 0.29 ± 0.019 | 0.23 ± 0.021 | 0.71 ± 0.019 | 0.77 ± 0.021 | 0.32 ± 0.017 | 0.95 ± 0.004 | 0.74 ± 0.005 | 0.82 ± 0.008 |
| **Test** | 0.18 ± 0.010 | 0.77 ± 0.013 | 0.28 ± 0.023 | 0.22 ± 0.018 | 0.72 ± 0.023 | 0.78 ± 0.018 | 0.32 ± 0.018 | 0.95 ± 0.005 | 0.75 ± 0.006 | 0.83 ± 0.008 |

**Table S5.** Summarized statistical characteristics of Hershberger classificatory models**.**

| **Set** | **Threshold ± SD** | **ACC ± SD** | **FNR ± SD** | **FPR ± SD** | **Recall ± SD** | **SP ± SD** | **PPV ± SD** | **NPV ± SD** | **G-mean ± SD** | **AUC ± SD** |
| --- | --- | --- | --- | --- | --- | --- | --- | --- | --- | --- |
| **MPNN** | | | | | | | | | | |
| **Training** | 0.50 ± 0.000 | 0.94 ± 0.006 | 0.41 ± 0.047 | 0.03 ± 0.011 | 0.59 ± 0.047 | 0.97 ± 0.011 | 0.66 ± 0.075 | 0.96 ± 0.004 | 0.76 ± 0.026 | 0.92 ± 0.011 |
| **Validation** | 0.50 ± 0.000 | 0.95 ± 0.014 | 0.32 ± 0.064 | 0.02 ± 0.016 | 0.68 ± 0.064 | 0.98 ± 0.016 | 0.74 ± 0.118 | 0.97 ± 0.005 | 0.82 ± 0.038 | 0.96 ± 0.008 |
| **Test** | 0.50 ± 0.000 | 0.96 ± 0.013 | 0.28 ± 0.056 | 0.02 ± 0.017 | 0.72 ± 0.056 | 0.98 ± 0.017 | 0.84 ± 0.149 | 0.97 ± 0.005 | 0.84 ± 0.031 | 0.95 ± 0.039 |
| **MPNN (calibrated)** | | | | | | | | | | |
| **Training** | 0.18 ± 0.054 | 0.89 ± 0.029 | 0.20 ± 0.033 | 0.10 ± 0.034 | 0.80 ± 0.033 | 0.90 ± 0.034 | 0.42 ± 0.079 | 0.98 ± 0.003 | 0.85 ± 0.007 | 0.92 ± 0.012 |
| **Validation** | 0.13 ± 0.058 | 0.88 ± 0.012 | 0.00 ± 0.000 | 0.13 ± 0.013 | 1.00 ± 0.000 | 0.87 ± 0.013 | 0.41 ± 0.024 | 1.00 ± 0.000 | 0.93 ± 0.007 | 0.96 ± 0.008 |
| **Test** | 0.13 ± 0.058 | 0.87 ± 0.066 | 0.28 ± 0.056 | 0.12 ± 0.069 | 0.72 ± 0.056 | 0.88 ± 0.069 | 0.41 ± 0.239 | 0.97 ± 0.008 | 0.80 ± 0.056 | 0.95 ± 0.039 |
| **GAT** | | | | | | | | | | |
| **Training** | 0.50 ± 0.000 | 0.95 ± 0.006 | 0.55 ± 0.162 | 0.01 ± 0.009 | 0.45 ± 0.162 | 0.99 ± 0.009 | 0.80 ± 0.075 | 0.96 ± 0.013 | 0.67 ± 0.112 | 0.93 ± 0.007 |
| **Validation** | 0.50 ± 0.000 | 0.98 ± 0.005 | 0.20 ± 0.078 | 0.01 ± 0.007 | 0.80 ± 0.078 | 0.99 ± 0.007 | 0.94 ± 0.078 | 0.98 ± 0.007 | 0.89 ± 0.042 | 0.98 ± 0.003 |
| **Test** | 0.50 ± 0.000 | 0.95 ± 0.010 | 0.46 ± 0.143 | 0.01 ± 0.017 | 0.54 ± 0.143 | 0.99 ± 0.017 | 0.92 ± 0.149 | 0.96 ± 0.013 | 0.73 ± 0.090 | 0.97 ± 0.014 |
| **GAT (calibrated)** | | | | | | | | | | |
| **Training** | 0.05 ± 0.021 | 0.86 ± 0.025 | 0.12 ± 0.008 | 0.14 ± 0.008 | 0.88 ± 0.008 | 0.86 ± 0.027 | 0.36 ± 0.053 | 0.99 ± 0.001 | 0.87 ± 0.015 | 0.93 ± 0.007 |
| **Validation** | 0.08 ± 0.075 | 0.92 ± 0.045 | 0.03 ± 0.064 | 0.09 ± 0.054 | 0.97 ± 0.064 | 0.91 ± 0.054 | 0.53 ± 0.252 | 1.00 ± 0.006 | 0.94 ± 0.014 | 0.98 ± 0.003 |
| **Test** | 0.08 ± 0.075 | 0.91 ± 0.050 | 0.13 ± 0.088 | 0.09 ± 0.062 | 0.87 ± 0.088 | 0.91 ± 0.062 | 0.54 ± 0.166 | 0.99 ± 0.009 | 0.89 ± 0.024 | 0.97 ± 0.014 |
| **GIN** | | | | | | | | | | |
| **Training** | 0.50 ± 0.000 | 1.00 ± 0.003 | 0.05 ± 0.039 | 0.00 ± 0.001 | 0.95 ± 0.039 | 1.00 ± 0.001 | 1.00 ± 0.008 | 1.00 ± 0.003 | 0.97 ± 0.020 | 1.00 ± 0.001 |
| **Validation** | 0.50 ± 0.000 | 0.96 ± 0.005 | 0.46 ± 0.064 | 0.00 ± 0.000 | 0.54 ± 0.064 | 1.00 ± 0.000 | 1.00 ± 0.000 | 0.96 ± 0.005 | 0.73 ± 0.045 | 0.98 ± 0.015 |
| **Test** | 0.50 ± 0.000 | 0.94 ± 0.015 | 0.62 ± 0.137 | 0.01 ± 0.007 | 0.38 ± 0.137 | 0.99 ± 0.007 | 0.89 ± 0.137 | 0.94 ± 0.013 | 0.62 ± 0.105 | 0.96 ± 0.036 |
| **GIN (calibrated)** | | | | | | | | | | |
| **Training** | 0.03 ± 0.044 | 1.00 ± 0.004 | 0.00 ± 0.008 | 0.00 ± 0.005 | 1.00 ± 0.008 | 1.00 ± 0.005 | 0.95 ± 0.049 | 1.00 ± 0.001 | 1.00 ± 0.004 | 1.00 ± 0.001 |
| **Validation** | 0.05 ± 0.048 | 0.96 ± 0.015 | 0.03 ± 0.064 | 0.05 ± 0.021 | 0.97 ± 0.064 | 0.95 ± 0.021 | 0.62 ± 0.128 | 1.00 ± 0.006 | 0.96 ± 0.024 | 0.98 ± 0.015 |
| **Test** | 0.05 ± 0.048 | 0.92 ± 0.035 | 0.12 ± 0.000 | 0.07 ± 0.039 | 0.88 ± 0.000 | 0.93 ± 0.039 | 0.51 ± 0.074 | 0.99 ± 0.001 | 0.90 ± 0.019 | 0.96 ± 0.036 |
| **AttentiveFP** | | | | | | | | | | |
| **Training** | 0.50 ± 0.000 | 0.95 ± 0.003 | 0.38 ± 0.087 | 0.02 ± 0.007 | 0.62 ± 0.087 | 0.98 ± 0.007 | 0.72 ± 0.042 | 0.97 ± 0.007 | 0.78 ± 0.053 | 0.93 ± 0.010 |
| **Validation** | 0.50 ± 0.000 | 0.95 ± 0.018 | 0.29 ± 0.101 | 0.03 ± 0.018 | 0.71 ± 0.101 | 0.97 ± 0.018 | 0.72 ± 0.135 | 0.97 ± 0.009 | 0.83 ± 0.060 | 0.96 ± 0.010 |
| **Test** | 0.50 ± 0.000 | 0.93 ± 0.019 | 0.46 ± 0.112 | 0.03 ± 0.019 | 0.54 ± 0.112 | 0.97 ± 0.019 | 0.64 ± 0.198 | 0.95 ± 0.011 | 0.72 ± 0.069 | 0.95 ± 0.021 |
| **AttentiveFP (calibrated)** | | | | | | | | | | |
| **Training** | 0.10 ± 0.040 | 0.86 ± 0.033 | 0.12 ± 0.039 | 0.14 ± 0.039 | 0.88 ± 0.039 | 0.86 ± 0.039 | 0.36 ± 0.049 | 0.99 ± 0.004 | 0.87 ± 0.012 | 0.93 ± 0.010 |
| **Validation** | 0.34 ± 0.085 | 0.94 ± 0.030 | 0.14 ± 0.000 | 0.05 ± 0.032 | 0.86 ± 0.000 | 0.95 ± 0.032 | 0.62 ± 0.167 | 0.99 ± 0.000 | 0.90 ± 0.015 | 0.96 ± 0.010 |
| **Test** | 0.34 ± 0.085 | 0.93 ± 0.023 | 0.36 ± 0.137 | 0.05 ± 0.033 | 0.64 ± 0.137 | 0.95 ± 0.033 | 0.63 ± 0.209 | 0.96 ± 0.013 | 0.78 ± 0.076 | 0.95 ± 0.021 |

**Table S6.** Summarized statistical characteristics of Uterotrophic classificatory models**.**

| **Set** | **Threshold ± SD** | **ACC ± SD** | **FNR ± SD** | **FPR ± SD** | **Recall ± SD** | **SP ± SD** | **PPV ± SD** | **NPV ± SD** | **G-mean ± SD** | **AUC ± SD** |
| --- | --- | --- | --- | --- | --- | --- | --- | --- | --- | --- |
| **MPNN** | | | | | | | | | | |
| **Training** | 0.50 ± 0.000 | 0.96 ± 0.002 | 0.22 ± 0.000 | 0.02 ± 0.002 | 0.78 ± 0.000 | 0.98 ± 0.002 | 0.75 ± 0.014 | 0.98 ± 0.000 | 0.87 ± 0.001 | 0.98 ± 0.002 |
| **Validation** | 0.50 ± 0.000 | 0.97 ± 0.005 | 0.36 ± 0.137 | 0.01 ± 0.006 | 0.64 ± 0.137 | 0.99 ± 0.006 | 0.78 ± 0.126 | 0.98 ± 0.007 | 0.79 ± 0.085 | 0.99 ± 0.002 |
| **Test** | 0.50 ± 0.000 | 0.93 ± 0.023 | 0.39 ± 0.099 | 0.03 ± 0.019 | 0.61 ± 0.099 | 0.97 ± 0.019 | 0.72 ± 0.170 | 0.96 ± 0.011 | 0.77 ± 0.069 | 0.97 ± 0.014 |
| **MPNN (calibrated)** | | | | | | | | | | |
| **Training** | 0.02 ± 0.009 | 0.94 ± 0.016 | 0.06 ± 0.014 | 0.06 ± 0.018 | 0.94 ± 0.014 | 0.94 ± 0.018 | 0.55 ± 0.060 | 0.99 ± 0.001 | 0.94 ± 0.006 | 0.98 ± 0.002 |
| **Validation** | 0.23 ± 0.068 | 0.99 ± 0.005 | 0.00 ± 0.000 | 0.01 ± 0.006 | 1.00 ± 0.000 | 0.99 ± 0.006 | 0.77 ± 0.060 | 1.00 ± 0.000 | 0.99 ± 0.003 | 0.99 ± 0.002 |
| **Test** | 0.23 ± 0.068 | 0.95 ± 0.028 | 0.16 ± 0.061 | 0.04 ± 0.030 | 0.84 ± 0.061 | 0.96 ± 0.030 | 0.75 ± 0.172 | 0.98 ± 0.007 | 0.90 ± 0.037 | 0.97 ± 0.014 |
| **GAT** | | | | | | | | | | |
| **Training** | 0.50 ± 0.000 | 0.97 ± 0.004 | 0.27 ± 0.018 | 0.01 ± 0.004 | 0.73 ± 0.018 | 0.99 ± 0.004 | 0.80 ± 0.038 | 0.98 ± 0.001 | 0.85 ± 0.010 | 0.98 ± 0.004 |
| **Validation** | 0.50 ± 0.000 | 1.00 ± 0.000 | 0.00 ± 0.000 | 0.00 ± 0.000 | 1.00 ± 0.000 | 1.00 ± 0.000 | 1.00 ± 0.000 | 1.00 ± 0.000 | 1.00 ± 0.000 | 1.00 ± 0.000 |
| **Test** | 0.50 ± 0.000 | 0.94 ± 0.005 | 0.33 ± 0.000 | 0.02 ± 0.006 | 0.67 ± 0.000 | 0.98 ± 0.006 | 0.77 ± 0.048 | 0.96 ± 0.000 | 0.81 ± 0.002 | 0.96 ± 0.009 |
| **GAT (calibrated)** | | | | | | | | | | |
| **Training** | 0.07 ± 0.030 | 0.96 ± 0.001 | 0.08 ± 0.008 | 0.04 ± 0.001 | 0.92 ± 0.008 | 0.96 ± 0.001 | 0.66 ± 0.008 | 0.99 ± 0.001 | 0.94 ± 0.004 | 0.98 ± 0.004 |
| **Validation** | 0.39 ± 0.102 | 1.00 ± 0.000 | 0.00 ± 0.000 | 0.00 ± 0.000 | 1.00 ± 0.000 | 1.00 ± 0.000 | 1.00 ± 0.000 | 1.00 ± 0.000 | 1.00 ± 0.000 | 1.00 ± 0.000 |
| **Test** | 0.39 ± 0.102 | 0.94 ± 0.005 | 0.33 ± 0.000 | 0.02 ± 0.006 | 0.67 ± 0.000 | 0.98 ± 0.006 | 0.77 ± 0.048 | 0.96 ± 0.000 | 0.81 ± 0.002 | 0.96 ± 0.009 |
| **GIN** | | | | | | | | | | |
| **Training** | 0.50 ± 0.000 | 0.98 ± 0.002 | 0.20 ± 0.033 | 0.00 ± 0.001 | 0.80 ± 0.033 | 1.00 ± 0.001 | 0.99 ± 0.020 | 0.98 ± 0.002 | 0.89 ± 0.018 | 0.99 ± 0.003 |
| **Validation** | 0.50 ± 0.000 | 0.97 ± 0.006 | 0.46 ± 0.112 | 0.01 ± 0.007 | 0.54 ± 0.112 | 0.99 ± 0.007 | 0.80 ± 0.171 | 0.98 ± 0.005 | 0.73 ± 0.070 | 0.99 ± 0.003 |
| **Test** | 0.50 ± 0.000 | 0.93 ± 0.014 | 0.45 ± 0.079 | 0.02 ± 0.012 | 0.55 ± 0.079 | 0.98 ± 0.012 | 0.76 ± 0.132 | 0.95 ± 0.009 | 0.73 ± 0.053 | 0.97 ± 0.006 |
| **GIN (calibrated)** | | | | | | | | | | |
| **Training** | 0.05 ± 0.021 | 0.96 ± 0.009 | 0.03 ± 0.018 | 0.04 ± 0.011 | 0.97 ± 0.018 | 0.96 ± 0.011 | 0.64 ± 0.053 | 1.00 ± 0.001 | 0.96 ± 0.005 | 0.99 ± 0.004 |
| **Validation** | 0.08 ± 0.022 | 0.98 ± 0.005 | 0.00 ± 0.000 | 0.02 ± 0.006 | 1.00 ± 0.000 | 0.98 ± 0.006 | 0.69 ± 0.060 | 1.00 ± 0.000 | 0.99 ± 0.003 | 0.99 ± 0.003 |
| **Test** | 0.08 ± 0.022 | 0.94 ± 0.014 | 0.11 ± 0.000 | 0.05 ± 0.016 | 0.89 ± 0.000 | 0.95 ± 0.016 | 0.67 ± 0.064 | 0.99 ± 0.000 | 0.92 ± 0.008 | 0.97 ± 0.006 |
| **AttentiveFP** | | | | | | | | | | |
| **Training** | 0.50 ± 0.000 | 0.96 ± 0.003 | 0.15 ± 0.019 | 0.03 ± 0.003 | 0.85 ± 0.019 | 0.97 ± 0.003 | 0.72 ± 0.024 | 0.99 ± 0.001 | 0.91 ± 0.011 | 0.98 ± 0.004 |
| **Validation** | 0.50 ± 0.000 | 0.97 ± 0.013 | 0.16 ± 0.137 | 0.03 ± 0.007 | 0.84 ± 0.137 | 0.98 ± 0.007 | 0.67 ± 0.110 | 0.99 ± 0.007 | 0.91 ± 0.076 | 0.99 ± 0.003 |
| **Test** | 0.50 ± 0.000 | 0.96 ± 0.023 | 0.11 ± 0.000 | 0.03 ± 0.026 | 0.89 ± 0.000 | 0.97 ± 0.026 | 0.77 ± 0.149 | 0.99 ± 0.000 | 0.93 ± 0.012 | 0.97 ± 0.008 |
| **AttentiveFP (calibrated)** | | | | | | | | | | |
| **Training** | 0.01 ± 0.000 | 0.93 ± 0.013 | 0.06 ± 0.032 | 0.07 ± 0.017 | 0.94 ± 0.032 | 0.93 ± 0.017 | 0.50 ± 0.046 | 0.99 ± 0.003 | 0.93 ± 0.009 | 0.98 ± 0.003 |
| **Validation** | 0.13 ± 0.098 | 0.98 ± 0.012 | 0.00 ± 0.000 | 0.02 ± 0.012 | 1.00 ± 0.000 | 0.98 ± 0.012 | 0.67 ± 0.115 | 1.00 ± 0.000 | 0.99 ± 0.006 | 0.99 ± 0.003 |
| **Test** | 0.13 ± 0.098 | 0.95 ± 0.024 | 0.11 ± 0.000 | 0.05 ± 0.027 | 0.89 ± 0.000 | 0.95 ± 0.027 | 0.71 ± 0.130 | 0.99 ± 0.000 | 0.92 ± 0.013 | 0.97 ± 0.008 |

**REFERENCES**

1. U.S. EPA. 2025. ToxCast & Tox21 Summary Files from invitrodb_v4_3. Retrieved from <https://www.epa.gov/chemical-research/toxicity-forecaster-toxcasttm-data> on August 29, 2025. Data released August 2025.
2. Geisinger KR, Kute TE, Pettenati MJ, et al. Characterization of a human ovarian carcinoma cell line with estrogen and progesterone receptors. *Cancer*. 1989;63(2):280-288. doi:10.1002/1097-0142(19890115)63:2<280::aid-cncr2820630213>3.0.co;2-n
3. Rogers JM, Denison MS. Recombinant cell bioassays for endocrine disruptors: development of a stably transfected human ovarian cell line for the detection of estrogenic and anti-estrogenic chemicals. *In Vitr Mol Toxicol*. 2000;13(1):67-82.
4. OECD (2021), *Test No. 455: Performance-Based Test Guideline for Stably Transfected Transactivation In Vitro Assays to Detect Estrogen Receptor Agonists and Antagonists*, OECD Guidelines for the Testing of Chemicals, Section 4, OECD Publishing, Paris, <https://doi.org/10.1787/9789264265295-en>.
